# Unveiling the regioselectivity of rhodium(I)-catalyzed [2 + 2 + 2] cycloaddition reactions for open-cage C_70_ production

**DOI:** 10.3762/bjoc.20.28

**Published:** 2024-02-13

**Authors:** Cristina Castanyer, Anna Pla-Quintana, Anna Roglans, Albert Artigas, Miquel Solà

**Affiliations:** 1 Institut de Química Computacional i Catàlisi (IQCC) and Departament de Química, Universitat de Girona (UdG), Girona 17003 Catalunya, Spain

**Keywords:** cycloadditions, DFT calculations, [70]fullerene, open-cage fullerenes, rhodium

## Abstract

The regioselective functionalization of fullerenes holds significant promise for applications in the fields of medicinal chemistry, materials science, and photovoltaics. In this study, we investigate the regioselectivity of the rhodium(I)-catalyzed [2 + 2 + 2] cycloaddition reactions between diynes and C_70_ as a novel procedure for generating C_70_ bis(fulleroid) derivatives. The aim is to shed light on the regioselectivity of the process through both experimental and computational approaches. In addition, the photooxidation of one of the C–C double bonds in the synthesized bis(fulleroids) affords open-cage C_70_ derivatives having a 12-membered ring opening.

## Introduction

The discovery of C_60_ (buckminsterfullerene) in 1985 [1] initiated the search for possible technological applications of fullerenes. Nowadays, applications for these carbon-based molecules have been proposed in different fields such as medicinal chemistry [2–6], materials science [7–8], energy production, storage, and delivery [9–13], and electronics and optoelectronics [14–16]. Despite fullerenes having immense promise in all of these areas, their practical applications are still in various stages of research and development.

The functionalization of fullerenes makes them versatile materials, broadening the range of potential applications [17–18]. It allows the properties of these carbon cages to be tuned, making them more soluble (especially in water for medical applications) and improving their stability, among other desirable properties. The most common reactions used to functionalize fullerenes are Diels–Alder and 1,3-dipolar cycloadditions and Bingel–Hirsch cyclopropanations [19–20].

In most cases, functionalization occurs while preserving the carbon cages. However, in other cases, some of the bonds between the C atoms of the cage are broken and the cage is opened. The first example of an open-cage fullerene was reported in 1995 by Hummelen, Prato, and Wudl [21] through the reaction of C_60_ with azides followed by photooxygenation. Since then, many open-cage C_60_ derivatives have been reported. These open-cage fullerenes can act as molecular containers. Of special interest is the procedure called molecular surgery designed by Murata et al. [22–25] in which a hole in the fullerene is opened, an atom or small molecule is introduced and then the hole is closed restoring the original cage. Among the species that have been incarcerated with this procedure, we can find He, Ne, Ar, Kr, H_2_, N_2_, O_2_, HF, CO, CO_2_, H_2_O, H_2_O_2_, CH_4_, NH_3_, HCOH, HCCH, and CH_3_OH [26–27]. The encapsulation of atoms or small molecules inside the fullerene has been found to be able to produce meaningful changes in the reactivity of the cage [28–32].

**Scheme 1:**
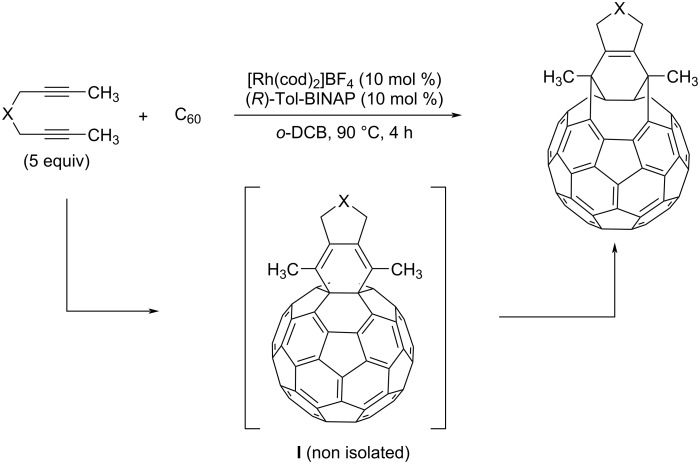
Rhodium(I)-catalyzed cycloaddition of C_60_ with diynes to afford bis(fulleroid) derivatives [33].

**Figure 1:**
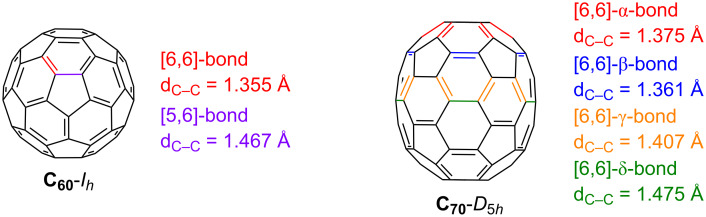
Types of [6,6]-bonds together with the [5,6]-bond of C_60_ with their C–C distances in pristine C_60_ and C_70_.

In 2018, our group reported a catalytic process to transform C_60_ in bis(fulleroid) derivatives [33–35]. This transformation encompassed a partially intermolecular Rh-catalyzed [2 + 2 + 2] cycloaddition reaction between diynes and C_60_, followed by a cage-opening through a Rh‐catalyzed di‐π‐methane rearrangement (Scheme 1). It is well-known that [6,6]-bonds (the bonds at the junction between two six-membered rings, Figure 1, left) are more reactive than [5,6]-bonds in C_60_ [36–38], and, not unexpectedly, the [6,6]-bond in C_60_ was the one involved in this [2 + 2 + 2] cycloaddition.

Although there are several papers reporting the opening of a hole in C_70_ [39–45], this chemistry has been less explored than in C_60_. The lower *D*_5_*_h_* symmetry of C_70_ compared to the *I_h_* of C_60_ increases the number of possible regioisomers. Indeed, C_70_ has eight different bonds, half of which are different types of [6,6]-bonds, namely the α-, β-, γ-, and δ-bonds (Figure 1, right) [46]. The α- and β-bonds of C_70_ are the most reactive ones [47].

With this in mind, the main goal of the present work is to explore, both experimentally and computationally, the Rh-catalyzed intermolecular [2 + 2 + 2] cycloaddition reaction between diynes and C_70_ as a new procedure to generate C_70_ bis(fulleroids). We are particularly interested in the analysis of the regioselectivity of this [2 + 2 + 2] cycloaddition.

## Results and Discussion

**Scheme 2:**
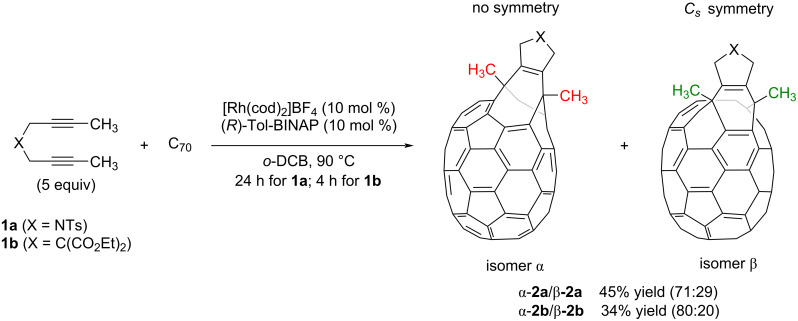
Rhodium-catalyzed cycloaddition of C_70_ with diynes **1a** and **1b**.

We started our study by testing the cycloaddition of *N*-tosyl-tethered bisalkyne **1a** and C_70_ (Scheme 2) using our previously optimized reaction conditions for the C_60_ derivative [33]: that is, using 10 mol % of a mixture of [Rh(cod)_2_]BF_4_ and Tol-BINAP in *o*-dichlorobenzene (*o*-DCB) and heating at 90 °C for 4 hours. The crude reaction mass obtained with these conditions was then purified by column chromatography (toluene). After eluting unreacted pristine C_70_, a dark reddish fraction was isolated and analyzed by HPLC. A major peak was observed at a retention time of 17.5 minutes, which we analyzed by UV–vis spectroscopy. This peak was assigned as a bis(fulleroid) compound by comparing the spectra with the UV–vis absorption pattern exhibited by previously characterized C_70_ bis(fulleroids) reported by Murata et al. [43,48]. In addition, a minor peak at a retention time of 20 minutes was also observed in the HPLC chromatogram, whose UV–vis has a pattern that is similar to a previously reported α-adduct [49]. We reasoned that this minor compound was the cyclohexadiene-fused C_70_ intermediate, analogous to cyclohexadiene-fused C_60_ **I** (see Scheme 1), which had not completely evolved into the corresponding bis(fulleroid) product after 4 h of reaction (Figure S1 in Supporting Information File 1). Importantly, the observation of this intermediate represents an experimental proof of the proposed reaction mechanism. Confirmation that only one unit of **1a** reacted with C_70_ in the reaction was obtained from HRMS, which gave a single peak at *m*/*z* 1138.0868 corresponding to [**2a** + Na]^+^. Further optimization was then carried out to obtain the bis(fulleroid) derivative alone (Table S1 in Supporting Information File 1). On increasing the reaction temperature to 120 °C and 180 °C the results were found to be the same, showing that 90 °C is sufficient for the reaction to proceed. In contrast, on extending the reaction time to 24 hours, the minor peak in the HPLC disappeared and only the peak corresponding to the bis(fulleroid) remained. The yield of derivative **2a** was 45%. Other experiments were run using other solvents such as toluene and chlorobenzene, increasing the C_70_ concentration from 1.2 M to 2.4 M, and decreasing the catalytic load to 5 mol % (Table S1 in Supporting Information File 1). However, none of these trials improved the yield of bis(fulleroid) **2a**.

The same reaction was run starting with malonate-tethered diyne **1b**. In this case, the reaction was finished after 4 hours and bis(fulleroid) **2b** was obtained with a 34% yield (Scheme 2). The corresponding compound **2b** was analyzed by HPLC, giving only one peak. UV–vis experiments revealed the formation of a bis(fulleroid) derivative (Figure S2 in Supporting Information File 1). The lower yield of **2b** compared to **2a** is probably due to the [2 + 2 + 2] homocoupling cycloaddition of the corresponding starting diyne, which is more favorable when the tether is a malonate rather than an NTs-sulfonamide.

**Figure 2:**
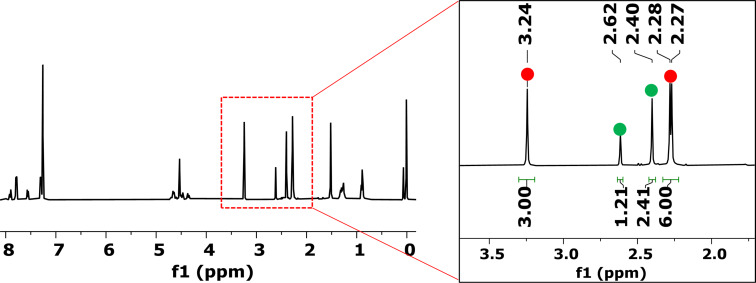
^1^H NMR (CS_2_/CDCl_3_, 400 MHz) spectrum of compound **2a** as a mixture of two isomers.

Among the four different [6,6]-bonds (α, β, γ, and δ) in pristine C_70_, α and β junctions are pyracylenic bonds, which happen to be the most reactive due to their higher degree of pyramidalization. Between both the α- and β-bonds, the higher curvature strain in α-bonds compared to β-double bonds makes the first one more reactive, leading to β-site isomers as minor products. Taking this into account, we carefully analyzed the NMR spectra of compound **2a**. Analysis of ^1^H NMR spectra of **2a** provided valuable information that confirmed the generation of two regioisomers in a 71:29 ratio (Figure 2). Comparable proportions of reactions at α- and β-bonds were systematically observed at different temperatures.

The protons of the two methyl groups around δ 2.30 ppm were used as diagnostic signals. For the major isomer (red dots, Figure 2), the spectrum exhibits two singlets at δ = 2.27 and 2.28 ppm, corresponding to the two different methyl groups in the six-membered ring formed in the cycloaddition and coming from the starting diyne **1a** (highlighted in red in Scheme 2). In contrast, for the minor isomer (green dots), which has *C_s_* symmetry, the two methyl groups (highlighted in green in Scheme 2) appear as a single peak at δ = 2.40 ppm. A second signal that also helps us to determine the ratio between the two isomers is the methyl of the tosyl group. For the major isomer the methyl appears at δ = 3.24 ppm, whereas for the minor isomer the peak is observed at δ = 2.62 ppm. Considering that isomer α has no symmetry and isomer β has *C_s_* symmetry [50], we can conclude that the major product formed was the α-isomer, as previously anticipated. All attempts to separate the two isomers by column chromatography and preparative TLC were unsuccessful. Malonate-tethered compound **2b** had the same spectroscopic behavior as **2a** though in this case the ratio in favor of the α-isomer was higher (80:20, Figure S6 in Supporting Information File 1).

**Figure 3:**
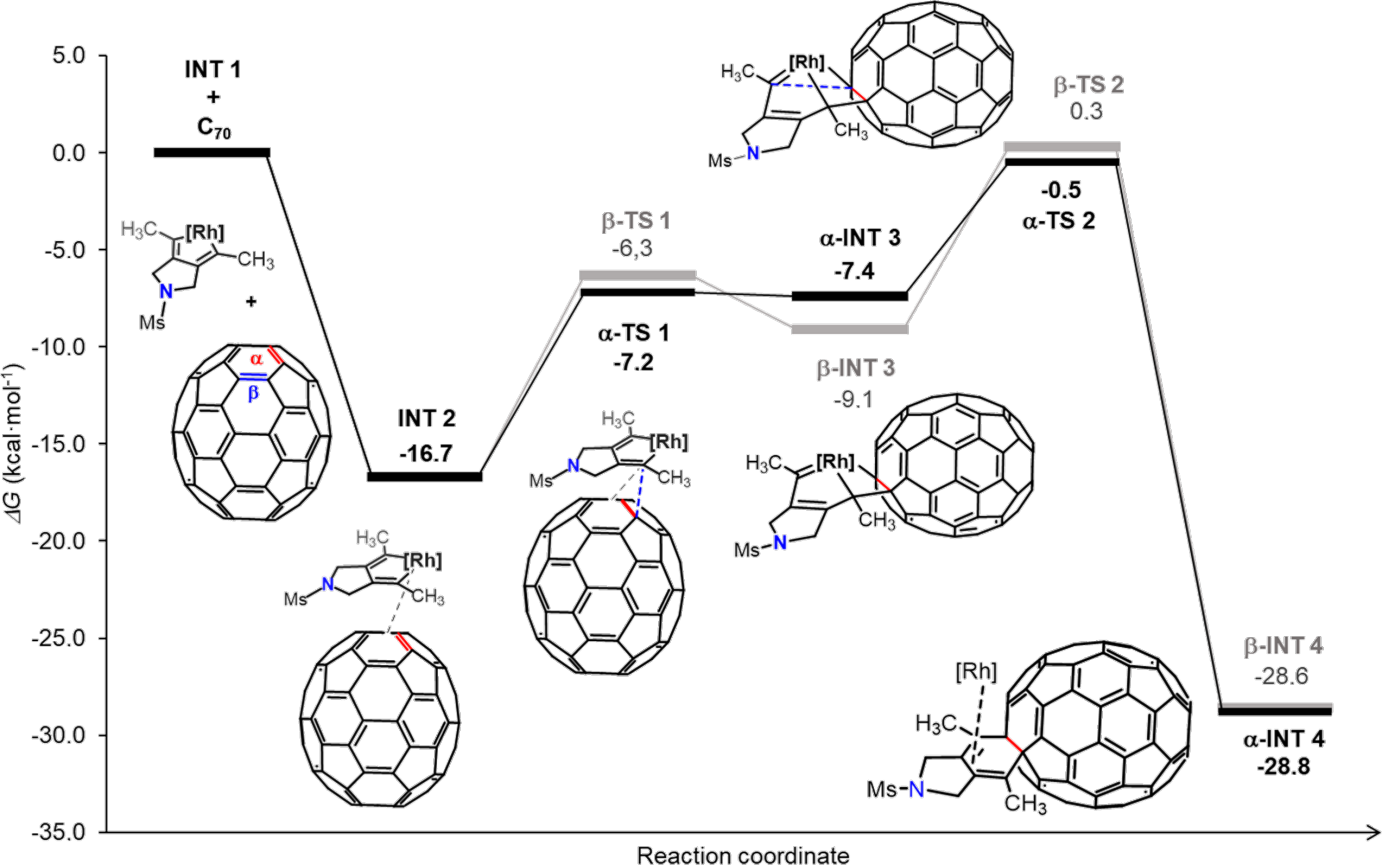
B3LYP-D3/cc-pVTZ-PP(SMD=o-DCB)//B3LYP-D3/CC-pVDZ-PP Gibbs energy profile of the [2 + 2 + 2] cycloaddition between our model diyne **1a** and C_70_. Comparison between α- and β-reaction pathways. Black line: α-pathway. Grey line: β-pathway. Molecular structures correspond to the α-pathway. [Rh] = [Rh(BIPHEP)]^+^.

To gain theoretical insight into the regioselectivity of the reaction, a density functional theory (DFT) investigation was carried out, as depicted in Figure 3. In the computations, the tosyl group was substituted by a mesyl substituent and BIPHEP was used as a model phosphine ligand instead of Tol-BINAP to reduce the computational cost. The calculations, conducted at the B3LYP-D3/cc-pVTZ-PP(SMD=o-DCB)//B3LYP-D3/cc-pVDZ-PP level (see full computational details in Supporting Information File 1), unveiled the following reaction mechanism: initially, an oxidative coupling of the two alkyne moieties of our model **1a** leads to the formation of **INT 1**, as previously reported [33]. This step, with a Gibbs energy barrier of 25.7 kcal·mol^−1^, is the rate-determining step for this process. Next, **INT 1** readily coordinates with a C_70_ molecule to generate **INT 2**, with this step being exergonic by 16.7 kcal·mol^−1^. From **INT 2**, the reaction can follow two distinct pathways, culminating in either an α-adduct or a β-adduct. In the α-adduct pathway (black line), a formal [2 + 2] cycloaddition occurs between the rhodacyclopentadiene moiety and a [6,6]-α-bond of C_70_, yielding rhodabicyclo[3.2.0]heptadiene intermediate α-**INT 3**. This step has a cost of 9.5 kcal·mol^−1^. Alternatively, a [6,6]-β-bond of C_70_ can be involved in this step (grey line) to produce β-**INT 3**, albeit with a slightly higher Gibbs energy barrier (ΔΔ*G* = 0.9 kcal·mol^−1^). The formation of intermediate α-**INT 3** and β-**INT 3** was found endergonic by 9.3 and 7.6 kcal·mol^−1^, respectively. Subsequently, both site isomers of **INT 3** can undergo reductive elimination with barriers of 6.9 and 9.4 kcal·mol^−1^ to deliver the corresponding cyclohexadiene-fused adducts, denoted as α-**INT 4** and β-**INT 4**, which will ultimately evolve into the final bis(fulleroid) reaction products [33]. As the site-selectivity of the reaction depends on these two consecutive steps, it indicates a preference for the α-bonds over the β-bonds, consistent with the experimental findings discussed earlier. Once **INT 1** is formed, for the rest of the process, the TOF determining transition state (TDTS) of the process is α/β-**TS 2** and the TOF determining intermediate (TDI) is **INT 2** and the energetic span (δ*G*) is 16.2 kcal·mol^−1^ for the α-attack and 17.0 kcal·mol^−1^ for the β-attack [51–52].

**Scheme 3:**
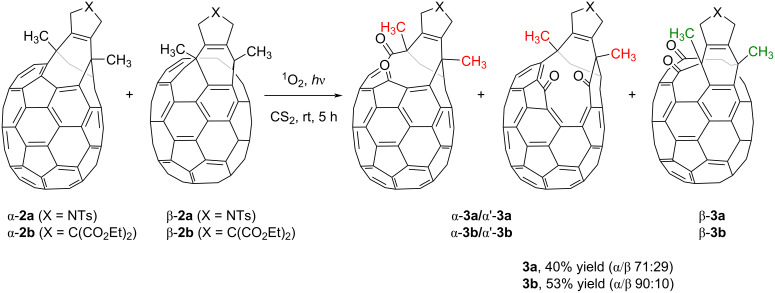
Oxidative cleavage of bis(fulleroid) derivatives **2a** and **2b**.

As previously described for our analogous C_60_ bis(fulleroids) [33], one of the double bonds of the eight-membered ring in **2a** can undergo oxidative cleavage affording open-cage C_70_ fullerenes that bear a twelve-membered orifice. There has been considerable interest in the construction of larger orifices in C_70_ derivatives given that the larger cavity compared to its C_60_ counterpart can facilitate the encapsulation of multiple atoms and molecules [40,43,53–54]. To fulfil this objective, compound **2a** was subjected to oxidative cleavage by exposing it to light in the presence of air (Scheme 3).

**Figure 4:**
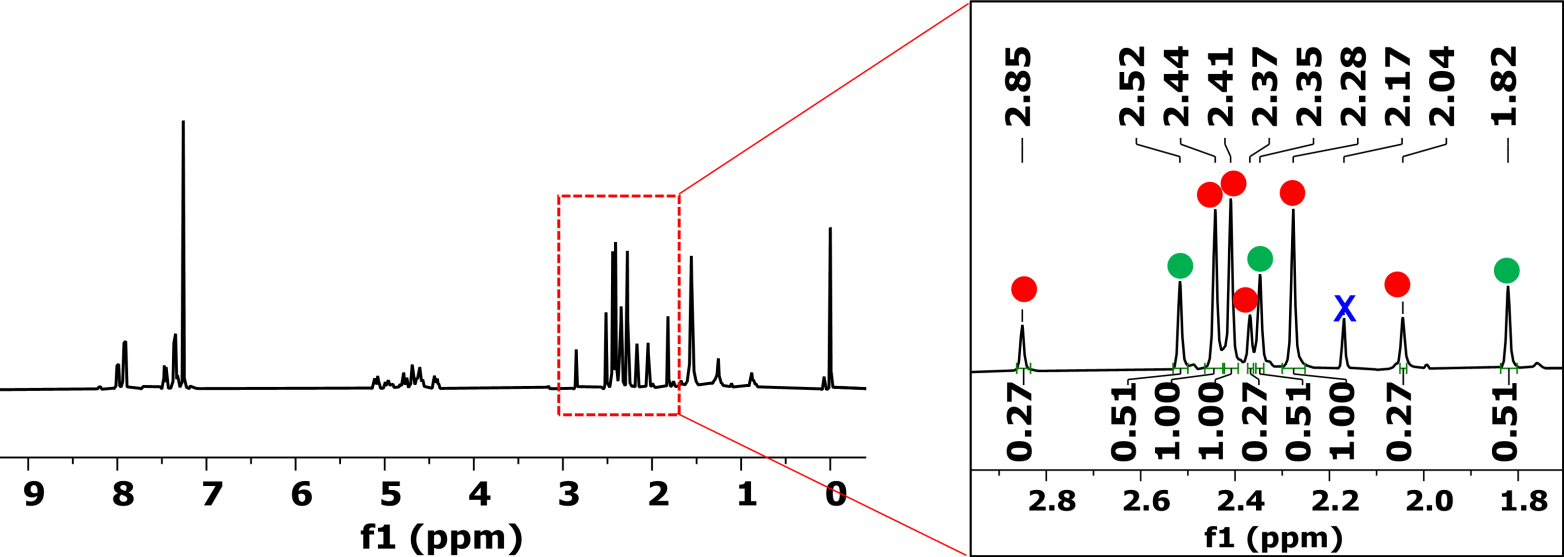
^1^H NMR (CS_2_/CDCl_3_, 400 MHz) spectrum of compound **3a** as a mixture of three isomers. X = residual toluene.

Given the lower symmetry of C_70_ in comparison to C_60_, the oxidative opening of the eight-membered ring of the mixture of α- and β-isomers **2** can result in more than two oxidized isomers, depending on which double bond is cleaved. After 5 hours of irradiation, the crude mixture was purified by column chromatography, giving an inseparable mixture of different isomers. The oxygenation process was confirmed by HRMS, which gave a single peak at *m*/*z* = 1170.0756 corresponding to [**3a** + Na]^+^. On analyzing carefully the mixture by ^1^H NMR spectroscopy, we observed three different sets of three methyl groups corresponding to the two methyls derived from bisalkyne **1a** and the methyl in the tosyl group in the spectrum (Figure 4). These results indicate that there are three regioisomers found in a ratio of 56:29:15. Two of them result from the oxidation of α-**2a**, whose lack of symmetry results in two different bonds available for oxidative cleavage. Site-isomer β-**2a** displays *C_s_* symmetry, and thus both bonds available for oxygenation are enantiotopic. Considering that starting **2a** consisted of a 71:29 mixture of α- and β-**2** isomers, we assumed that α- and α’-isomers (56% + 15% = 71%) correspond to the protons marked in red and the ones marked in green might be those of the β-isomer (29%). Unfortunately, NMR experiments did not allow to differentiate between α- and β-**3a** derivatives. The reaction was carried out also with bis(fulleroid) derivative **2b**, exhibiting the same behavior.

## Conclusion

In this study, we have explored the regioselectivity of the rhodium(I)-catalyzed [2 + 2 + 2] cycloaddition reaction between two different diynes and C_70_ with the objective of producing C_70_ bis(fulleroids). Mixtures of α- and β-site isomers were obtained, with the α-adduct being the major product of the reaction in both cases. This preference was rationalized by means of DFT calculations. Moreover, the photooxidation of one of the C–C double bonds of the new bis(fulleroids) afford open-cage C_70_ derivatives having a 12-membered ring opening. It is noteworthy to mention that examples of open-cage C_70_ derivatives are relatively scarce, likely owing to the challenges associated with their synthesis and the characterization of asymmetric structures. The findings of this study contribute to the ongoing efforts in the field of fullerene chemistry and provide a foundation for further exploration of regioselective [2 + 2 + 2] cycloaddition reactions as a means to tailor the properties of fullerenes for specific applications.

## Supporting Information

File 1 General materials and methods, experimental procedures and characterization of all new compounds.

## Data Availability

All experimental data available in published article and/or supplementary material. All computational data are available through the ioChem-BD repository: https://www.iochem-bd.org/handle/10/356060, https://doi.org/10.19061/iochem-bd-4-67.
