# Supplementary material for: Unveiling the regioselectivity of rhodium(I)-catalyzed [2 + 2 + 2] cycloaddition reactions for open-cage C70 production
Source: Beilstein J Org Chem. 2024 Feb 13;20:272–9. doi: 10.3762/bjoc.20.28 (PMC10877076; doi:10.3762/bjoc.20.28)
Supplement: File 1 — General materials and methods, experimental procedures and characterization of all new compounds. [file Beilstein_J_Org_Chem-20-272-s001.pdf]

BEILSTEIN JOURNAL OF ORGANIC CHEMISTRY

Supporting Information

for

Unveiling the regioselectivity of rhodium(I)-catalyzed [2 + 2 + 2] cycloaddition reactions for open-cage C_{70} production

Cristina Castanyer, Anna Pla-Quintana, Anna Roglans, Albert Artigas and Miquel Solà

Beilstein J. Org. Chem. **2024,** *20,* 272–279. doi:10.3762/bjoc.20.28

General materials and methods, experimental procedures and characterization of all new compounds

License and Terms: This is a supporting information file under the terms of the Creative Commons Attribution License (https://creativecommons.org/licenses/by/4.0). Please note that the reuse, redistribution and reproduction in particular requires that the author(s) and source are credited and that individual graphics may be subject to special legal provisions.
The license is subject to the *Beilstein Journal of Organic Chemistry* terms and conditions: (https://www.beilstein-journals.org/bjoc/terms)

Table of contents

General materials and methods S3

Table S1. Influence of the reaction conditions on the reaction outcome. S4

Figure S1. (a) HPLC trace of material (X = NTs) isolated after 16 h (entry 4 in Table S1); (b) HPLC trace of material isolated after 24 h (entry 5 in Table S1); (c) UV–vis spectra of $C_{70,}$ peak 1, peak 2 and previously reported alpha adduct [1]. S5

Figure S2. (a) HPLC trace of material (X = $C(COOEt)_2$) isolated after 4 h (entry 10 in Table S1; (b) UV–vis spectra of peak C_{70} and peak 1. .... S6

Scheme S1. Preparation and characterization of **2a**. S7

Figure S3. ^{1}H NMR spectrum (400 MHz, $CDCl_3/CS_2$) of compound **2a**. S8

Figure S4: MALDI-TOF HRMS spectrum of compound **2a**. S9

Figure S5. UV–vis spectrum (toluene) of compound **2a** (inset: HPLC trace of **2a**). S9

Scheme S2. Preparation and characterization of **2b** S10

Figure S6. ^{1}H NMR spectrum (400 MHz, $CDCl_3/CS_2$) of compound **2b**. S11

Figure S7. ^{13}C NMR spectrum (101 MHz, $CDCl_3$) of compound **2b**. S12

Figure S8. 2D-HSQC NMR spectrum of compound **2b**. S13

Figure S9. 2D-HMBC NMR spectrum of compound **2b**. S13

Figure S10. 2D-COSY NMR spectrum of compound **2b**. S14

Figure S11. MALDI-TOF HRMS spectrum of **2b**. S14

Figure S12. UV–vis spectrum ($CHCl_3$) of compound **2b** (inset: HPLC trace of **2b**). S15

Scheme S3. Oxidative cleavage of **2a**. S16

Figure S13. ^{1}H NMR spectrum (400 MHz, $CDCl_3/CS_2$) of compound **3a**. S17

Figure S14. ^{13}C NMR spectrum (101 MHz, $CDCl_3/CS_2$) of compound **3a**. S18

Figure S15. 2D-HSQC NMR spectrum of compound **3a**. S19

Figure S16. 2D-HMBC NMR spectrum of compound **3a**. S19

Figure S17. MALDI-TOF HRMS spectrum of compound **3a** (*m/z*). S20

Figure S18. UV–vis spectrum ($CHCl_3$) of compound **3a**. S21

Scheme S4. Preparation and characterization of **3b**. S22

Figure S19. ^{1}H NMR spectrum (400 MHz, $CDCl_3$) of **3b**. S23

Figure S20. ^{13}C NMR spectrum (101 MHz, $CDCl_3$) of compound **3b**. S24

Figure S21. 2D-HSQC NMR spectrum of compound **3b**. S25

Figure S22. 2D-HMBC NMR spectrum of compound **3b**. S25

Figure S23. MALDI-TOF HRMS spectrum of compound **3b**. S26

Figure S24. UV–vis spectrum ($CHCl_3$) of compound **3b**. S26

Computational details S27

Figure S25. Molecular structures of the two possible regioisomers of β-**2a** and their relative electronic energies computed at the B3LYP-D3/cc-pVDZ//B3LYP/cc-pVTZ level of theory.........S28

Figure S26. Molecular structure of (a) α-**TS 1** (b) α-**TS 2** (c) β-**TS 1** (d) β-**TS 2**...........................S29

References...S30

General materials and methods

Unless otherwise noted, materials were obtained from commercial suppliers and used without further purification. CH_2Cl_2 was dried under nitrogen by passing through solvent purification columns (MBraun, SPS-800). Reaction progress during the preparation of all compounds was monitored using thin layer chromatography on Macherey-Nagel Xtra SIL G/UV254 silica gel plates. Solvents were removed under reduced pressure with a rotary evaporator. Reaction mixtures were chromatographed on silica gel. All ^{1}H and ^{13}C NMR spectra were recorded on a Bruker ASCEND 400 spectrometer equipped with a 5 mm BBFO probe using $CDCl_3$ as a deuterated solvent. Chemical shifts for ^{1}H and ^{13}C NMR are reported in ppm (δ) relative to residual solvent signals. Coupling constants are given in hertz (Hz). ^{1}H and ^{13}C NMR signals were assigned based on 2D-NMR HSQC, HMBC and COSY experiments. Mass spectrometry analyses were recorded on a Bruker micrOTOF-Q II mass spectrometer (high resolution), equipped with electrospray ion source. The instrument was operated in the positive ESI(+) ion mode. HPLC data were collected on Agilent Technologies LC 1200 series instrument equipped with a Cosmosil Buckyprep-M column (10 mm × 250 mm, Nacalai Tesque, Inc.) monitored with a UV detector at 320 nm. Toluene was used as mobile phase (flow 0.5 mL/min). UV–vis spectra were performed with an Agilent 8452 UV–vis spectrophotometer (1 cm quartz cell) in toluene.

Table S1. Influence of the reaction conditions on the reaction outcome.

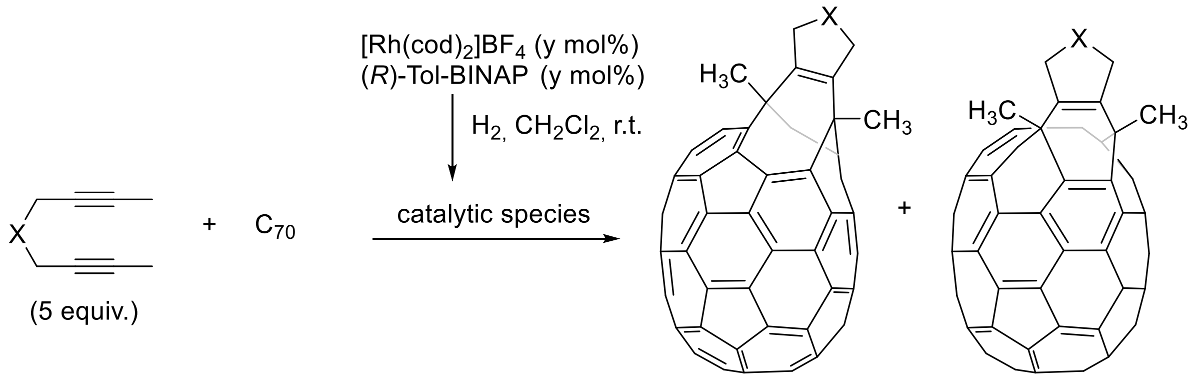

Entry	Solvent	y	X	[C_{70}] (mM)	Temperature (ºC)	Time (h)	Yield (%)	% *bis*(fulleroid)
**1**	*o*-DCB	10	NTs	1.2	90	4	46	90
**2**	*o*-DCB	10	NTs	1.2	120	4	42	90
**3**	*o*-DCB	10	NTs	1.2	180	4	42	90
**4**	*o*-DCB	10	NTs	1.2	90	16	45	95
**5**	*o*-DCB	10	NTs	1.2	90	24	45	>99
**6**	Toluene	10	NTs	1.2	90	24	22	>99
**7**	CB	10	NTs	1.2	90	24	28	>99
**8**	*o*-DCB	10	NTs	2.4	90	24	38	>99
**9**	*o*-DCB	5	NTs	1.2	90	24	30	>99
**10**	*o*-DCB	10	$C(COOEt)_2$	1.2	90	4	34	>99

o-DCB: *ortho*-Dichlorobenzene; CB: Chlorobenzene

Figure S1. (a) HPLC trace of material (X = NTs) isolated after 16 h (entry 4 in Table S1); (b) HPLC trace of material isolated after 24 h (entry 5 in Table S1); (c) UV–vis spectra of C_{70}, peak 1, peak 2 and previously reported alpha adduct [1].

a)

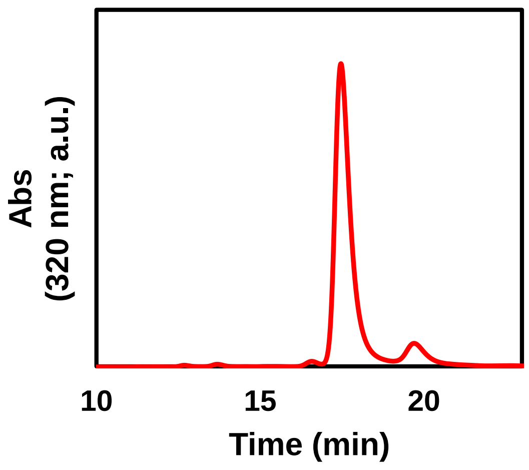

b)

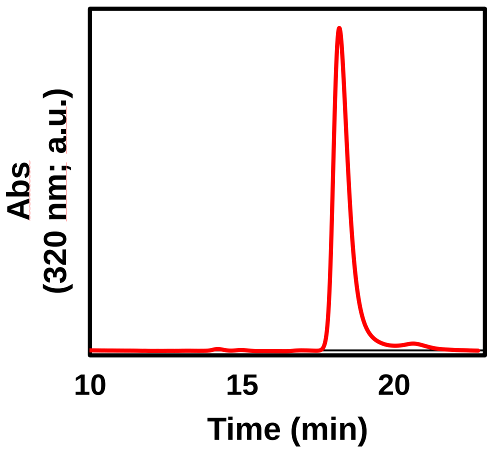

c)

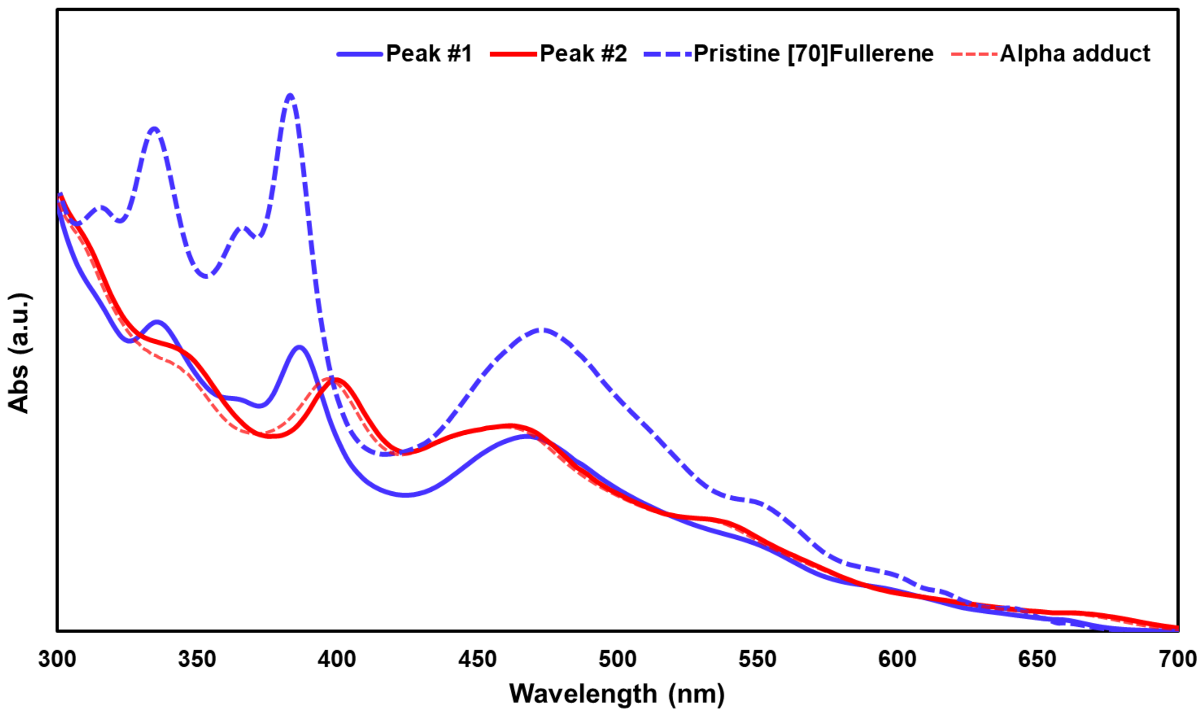

Figure S2. (a) HPLC trace of material (X = C(COOEt)$_2$) isolated after 4 h (entry 10 in Table S1; (b) UV–vis spectra of peak C_{70} and peak 1.

a)

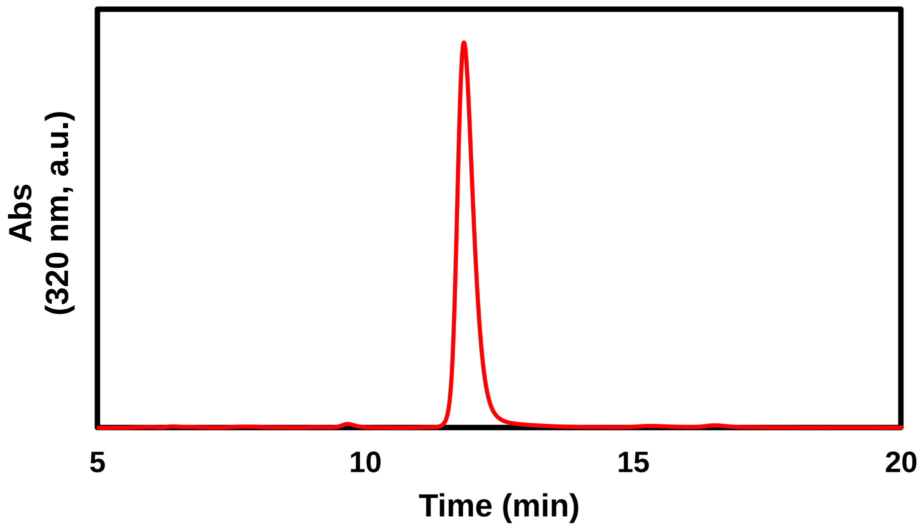

b)

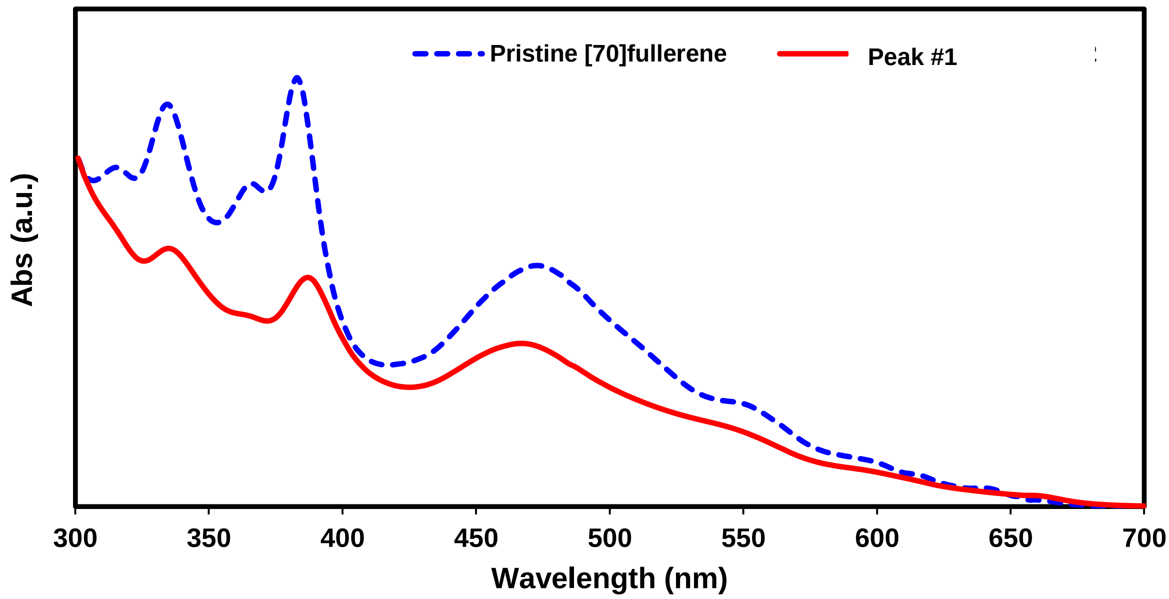

Scheme S1. Preparation and characterization of **2a**

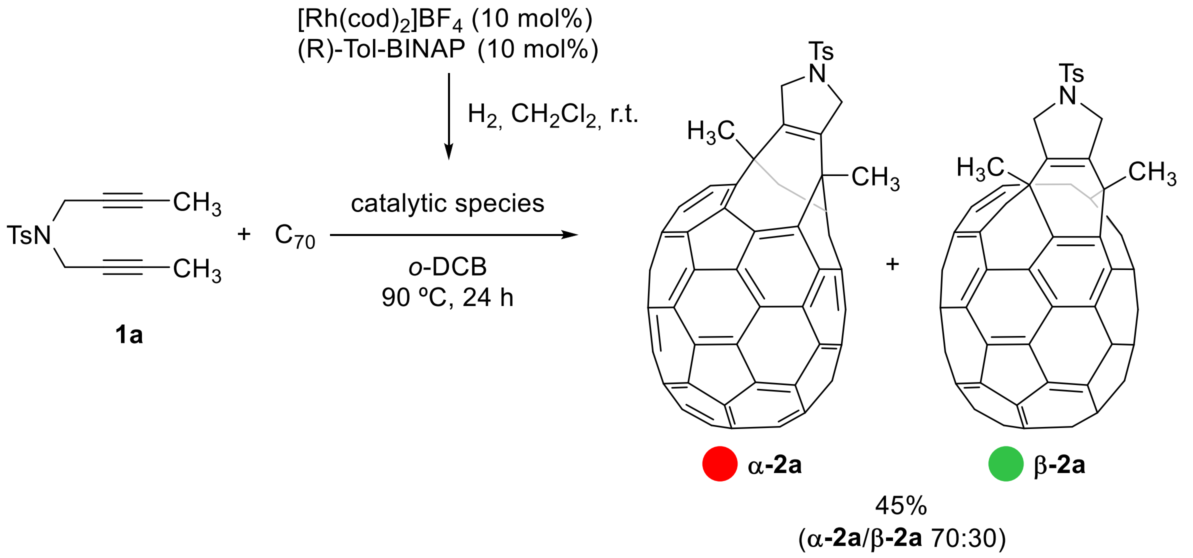

In a manner analogous to reference [2], in a 10 mL capped vial in an inert atmosphere, a solution of $[Rh(cod)_2]BF_4$ (2.4 mg, 0.006 mmol) and *(R)*-Tol-BINAP (4.1 mg, 0.006 mmol) in anhydrous CH_2Cl_2 (4 mL) was prepared. Hydrogen gas was bubbled into the catalyst solution for 30 min before it was concentrated to dryness, dissolved in anhydrous *o*-DCB and introduced *via* syringe into an *o*-DCB solution (1.2 mM) of C_{70} (50 mg, 0.06 mmol) and diyne **1a** (83 mg, 0.30 mmol) preheated to 90 °C. The resulting mixture was stirred at 90 °C overnight, allowed to cool to room temperature and concentrated under reduced pressure. The crude product was subjected to column chromatography (SiO_2, 40–63 μm, toluene) to provide **2a** (30 mg, 45%, **α-2a**/**β-2a** 70:30 as estimated by ^{1}H NMR integration) as a brown solid.

MW ($C_{85}H_{17}NO_2S$): 1115.1 g/mol; **R***f*: 0.28 (toluene); **^{1}H NMR (400 MHz, $CDCl_3/CS_2$) δ (ppm) α-2a:** 2.27 (s, 3H, C**H**$_3$−C), 2.28 (s, 3H, C**H**$_3$−C), 3.24 (s, 3H, C**H**$_3$−Ar), 4.33−4.70, m, 4H, C**H**$_2$−N), 7.30 (d, *J* = 8.0 Hz, 2H, C**H**−Ar), 7.79 (d, *J* = 8.0 Hz, 2H, C**H**−Ar); **β-2a:** 2.40 (s, 6H, C**H**$_3$−C), 2.62 (s, 3H, C**H**$_3$−Ar), 4.33−4.70, m, 4H, C**H**$_2$−N), 7.56 (d, *J* = 8.0, C−**H**$_{Ar}$), 7.91 (d, *J* = 8.0, C−**H**$_{Ar}$); **UV-vis (toluene) λ_{max} (nm)**: 337, 387, 467; **ESI-HRMS (*m/z*)** calcd for $[M+Na]^+$ = 1138.0872; found: 1138.0868.

Figure S3. ^{1}H NMR spectrum (400 MHz, $CDCl_3/CS_2$) of compound **2a**.

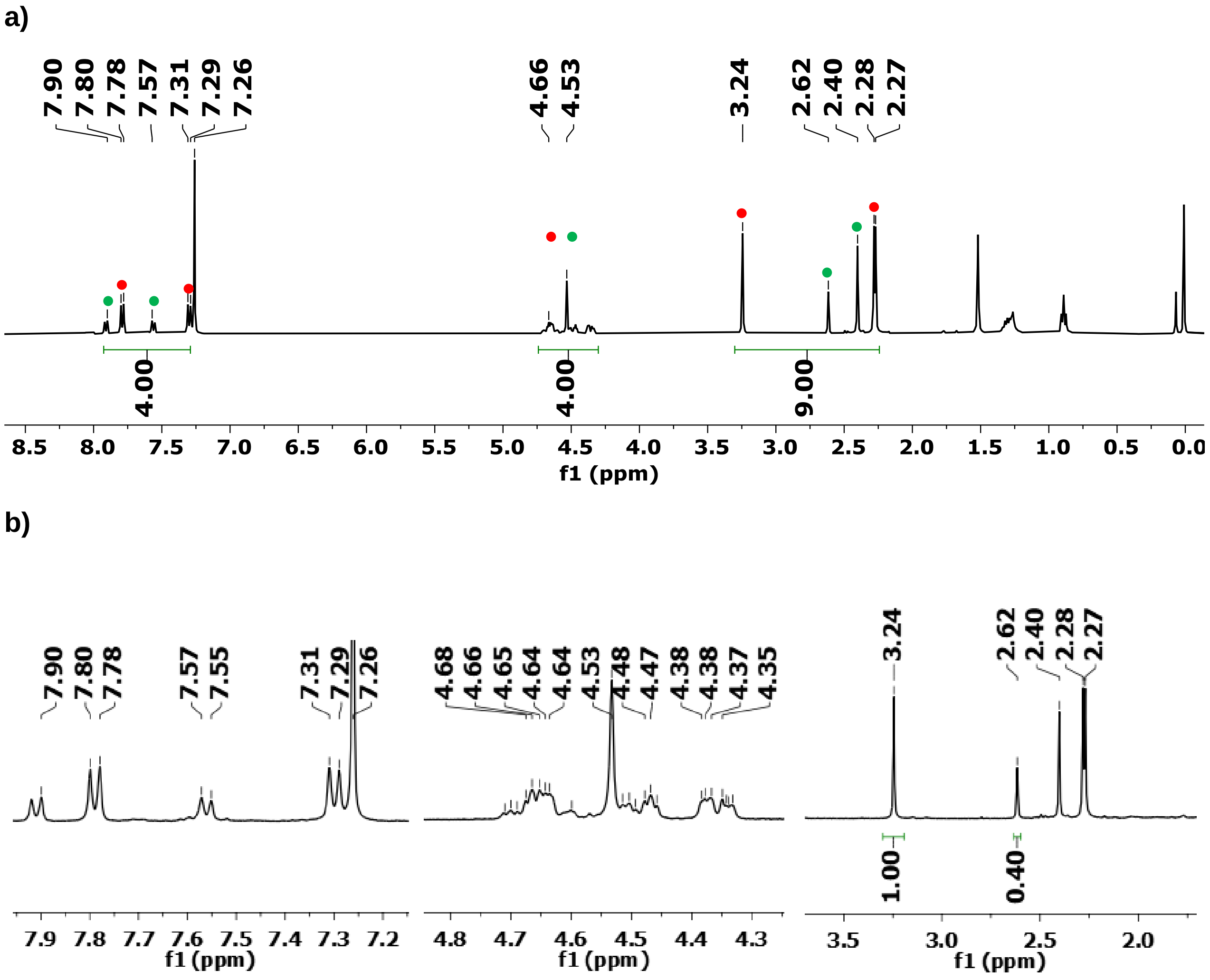

Figure S4: MALDI-TOF HRMS spectrum of compound **2a**.

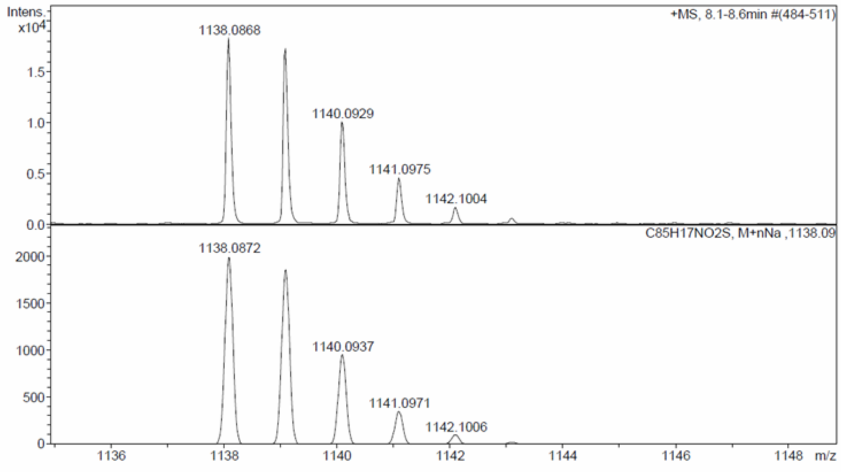

Figure S5. UV–vis spectrum (toluene) of compound **2a** (inset: HPLC trace of **2a**).

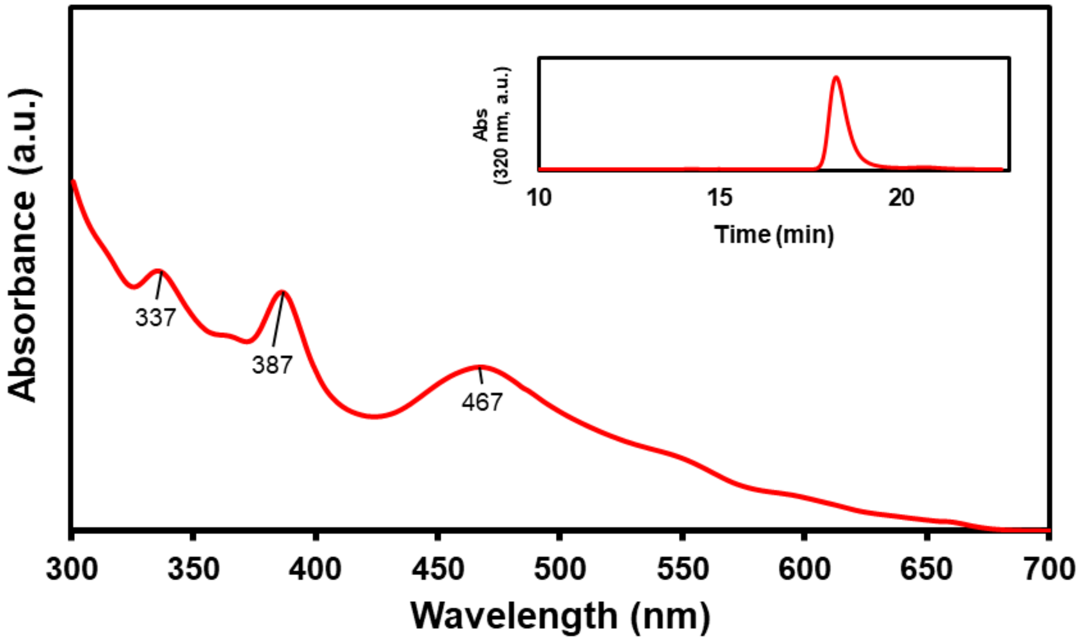

Scheme S2. Preparation and characterization of **2b**

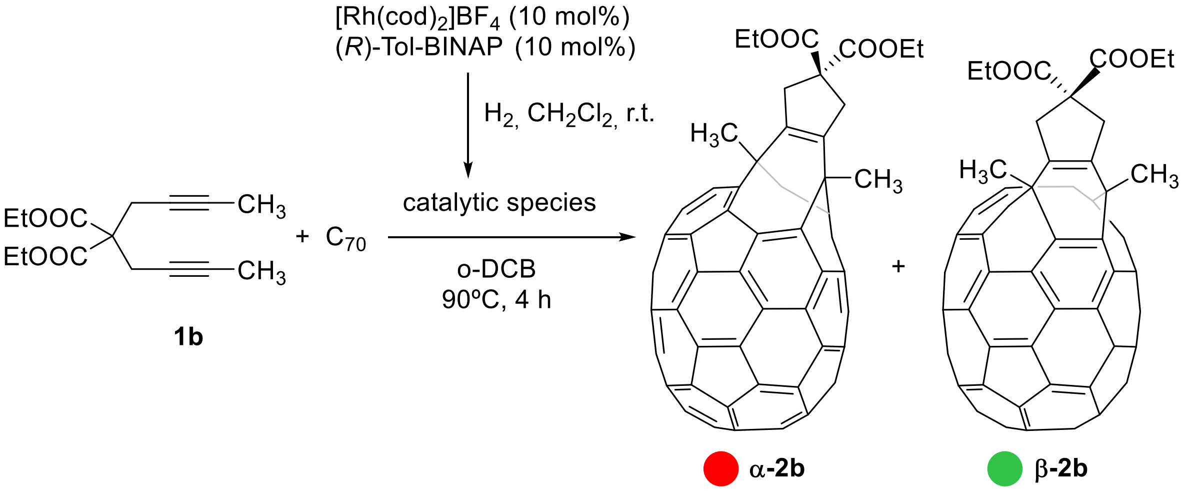

34%
(α-**2b**/β-**2b** 80:20)

In a manner analogous to reference [2], in a 10 mL capped vial in an inert atmosphere, a solution of [Rh(cod)$_2$]BF$_4$ (2.4 mg, 0.006 mmol) and *(R)*-Tol-BINAP (50 mg, 0.006 mmol) in anhydrous CH$_2$Cl$_2$ (4 mL) was prepared. Hydrogen gas was bubbled into the catalyst solution for 30 min before it was concentrated to dryness, dissolved in anhydrous *o*-DCB and introduced *via* syringe into an *o*-DCB solution (1.2 mM) of C$_{70}$ (50 mg, 0.06 mmol) and diyne **1b** (79 mg, 0.30 mmol) preheated to 90 °C. The resulting mixture was stirred at 90 °C for 4h, allowed to cool to room temperature and concentrated under reduced pressure. The crude product was subjected to column chromatography (SiO$_2$, 40–63 μm, toluene) to provide unreacted and **2b** (17 mg, 34%, **α-2b**/**β-2b** 80:20 as estimated by ^{1}H as a brown solid.

MW (C$_{85}$H$_{20}$O$_4$): g/mol; **R*f***: 0.56 (toluene); **^{1}H NMR (400 MHz, CDCl$_3$/CS$_2$) δ (ppm) α-2b:** 1.27 (t, *J* = 7.1 Hz, 3H, CH$_2$−C**H$_3$**), 1.35 (t, *J* = 7.1 Hz, 3H, CH$_2$−C**H$_3$**), 2.36 (s, 3H, C−C**H$_3$**), 3.23–3.62 (m, 4H, C-C**H$_2$**), 4.24 (q, *J* = 7.1 Hz, 2H, C**H$_2$**−CH$_3$), 4.33 (q, *J* = 7.1 Hz, 2H, C**H$_2$**−CH$_3$); **β-2b:** 1.35 (t, *J* = 7.1 Hz, 3H, CH$_2$−C**H$_3$**), 1.46 (t, *J* = 7.1 Hz, 3H, CH$_2$−C**H$_3$**), 2.48 (s, 6H, C−C**H$_3$**), 3.42 (s, 4H, C−C**H$_2$**), 4.43 (q, *J* = 7.1 Hz, 2H, C**H$_2$**−CH$_3$); one methylene signal overlapped; **^{13}C NMR (101 MHz, CDCl$_3$/CS$_2$) δ (ppm) α-2b** 14.29 (CH$_2$−**C**H$_3$), 14.33 (CH$_2$−**C**H$_3$), 27.00 (C−**C**H$_3$), 32.17 (C−**C**H$_3$), 40.24 (**C**−CH$_3$), 40.32 (C−**C**H$_2$), 40.39 (C-**C**H$_2$), 41.38 (**C**−CH$_3$); 58.32 (CH$_2$-**C**), 62.16 (**C**H$_2$-CH$_3$), 119.63−151.07 (**C**$_{quat}$), 171.54 (**C**=O), 171.86 (**C**=O); **β-2b:** 14.57 (CH$_2$−**C**H$_3$), 26.61 (C−**C**H$_3$), 40.02 (**C**-CH$_2$), 40.48 (C−**C**H$_2$), 40.39, 58.37 (CH$_2$-**C**−C=O), 62.33 (**C**H$_2$-CH$_3$), 119.63−151.07 (**C**$_{quat}$), 171.54 (**C**=O), 171.67 (**C**=O); **UV-vis (toluene) λ$_{max}$ (nm)**: 337, 387, 469; **ESI-HRMS (*m/z*)** calcd for [M+Na]$^+$ = 1127.1254; found: 1127.1243.

Figure S6. ^{1}H NMR spectrum (400 MHz, $CDCl_3/CS_2$) of compound **2b**.

a)

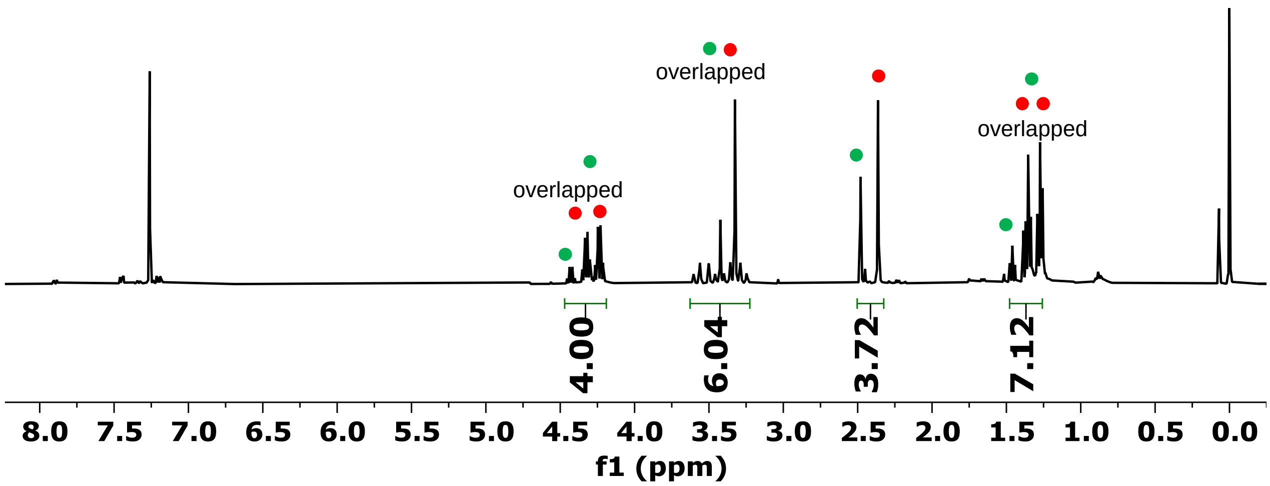

b)

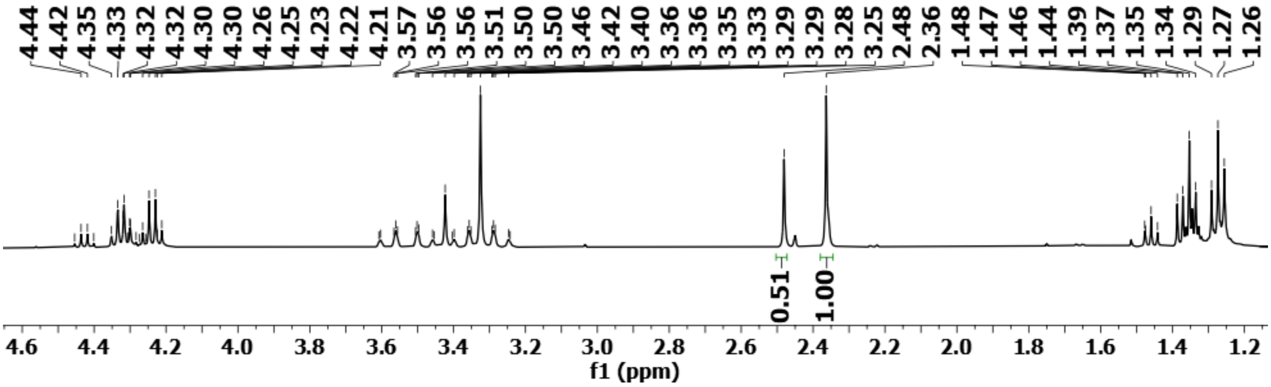

Figure S7. ^{13}C NMR spectrum (101 MHz, $CDCl_3$) of compound **2b**.

a)

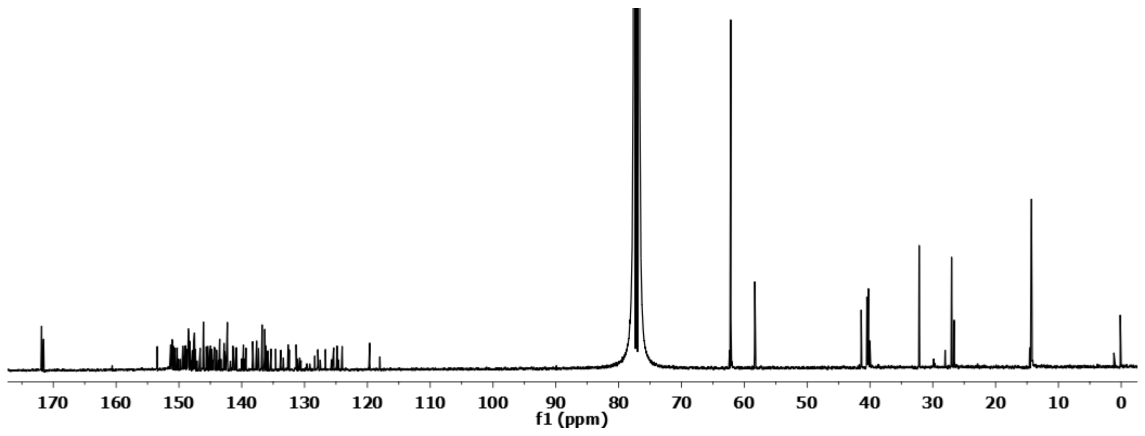

b)

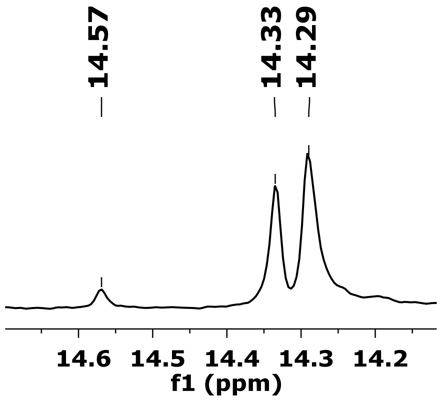

c)

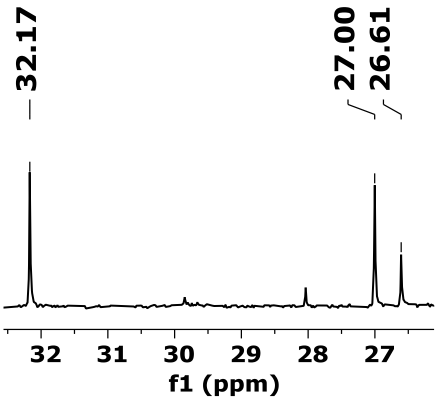

d)

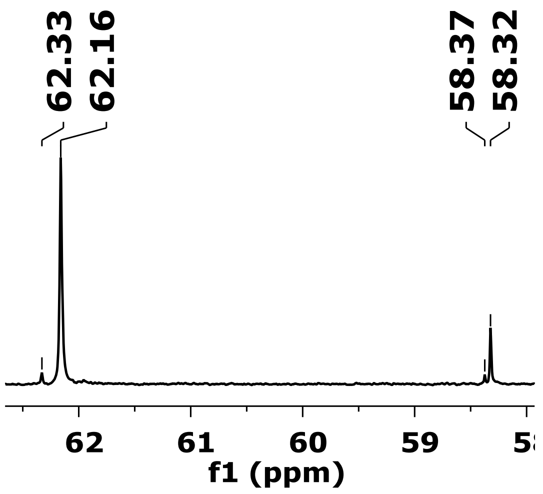

e)

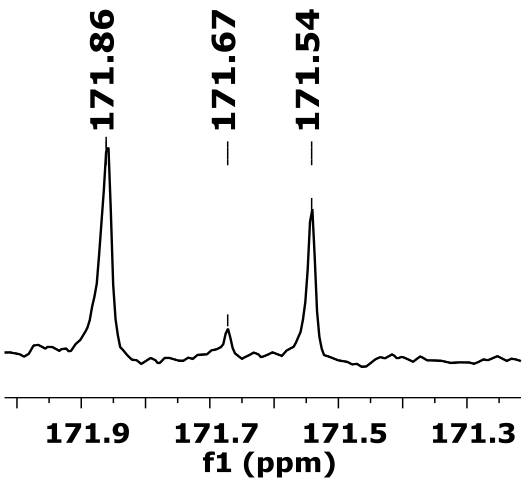

Figure S8. 2D-HSQC NMR spectrum of compound **2b**.

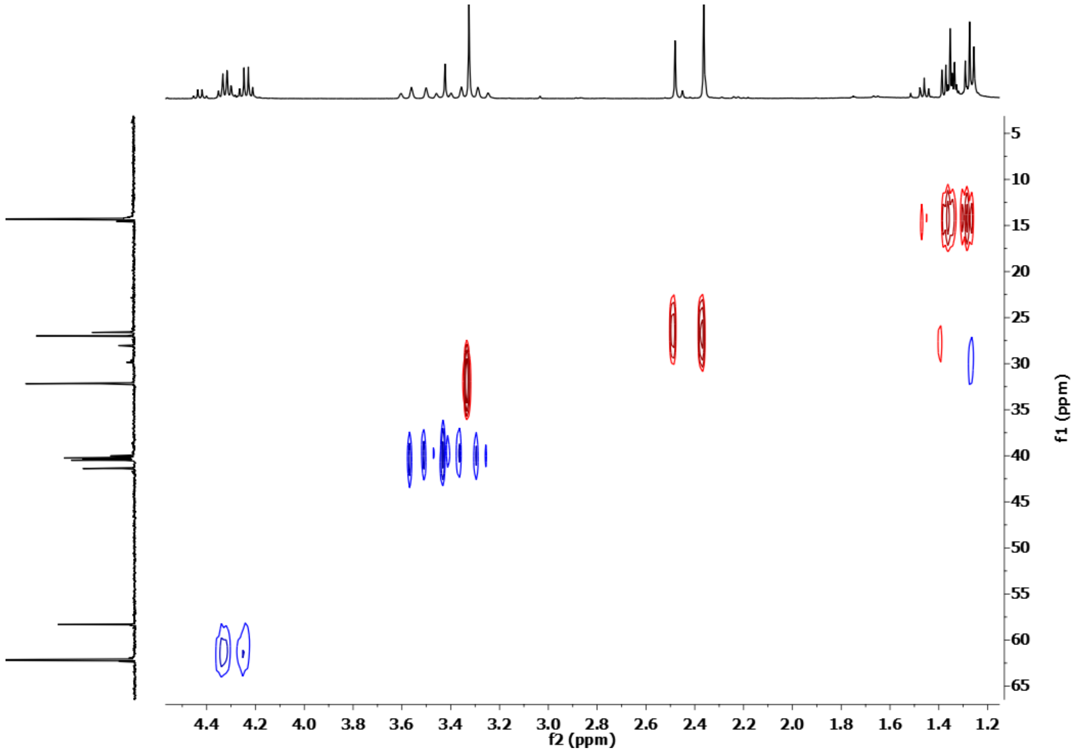

Figure S9. 2D-HMBC NMR spectrum of compound **2b**.

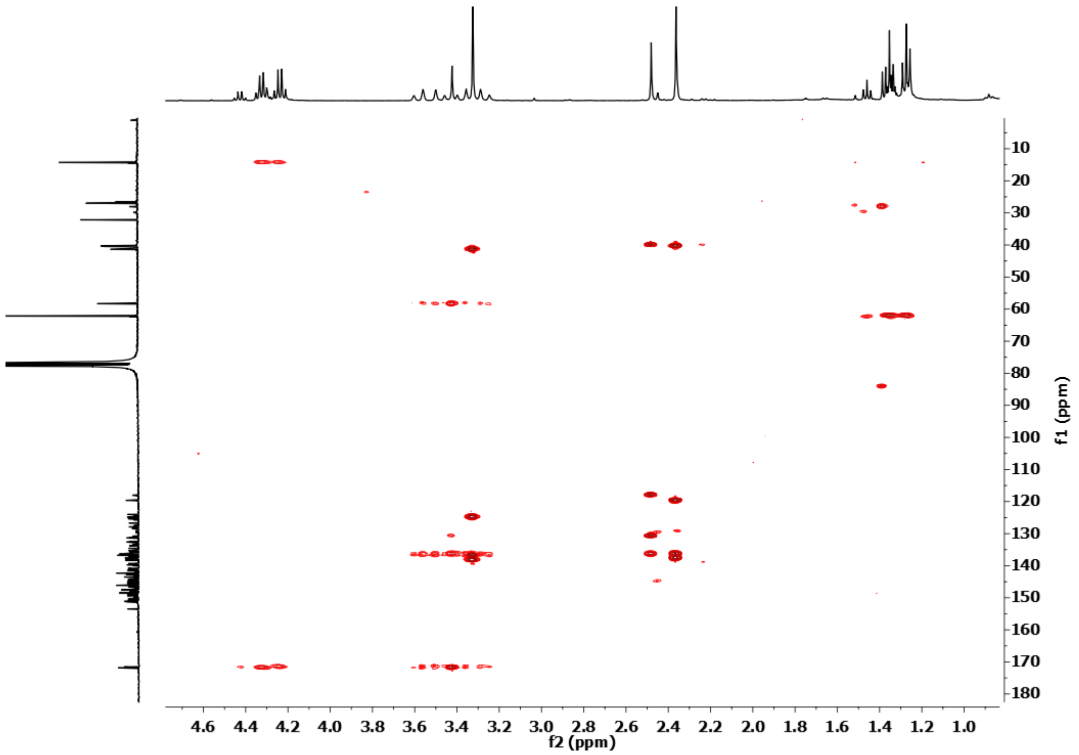

Figure S10. 2D-COSY NMR spectrum of compound **2b**.

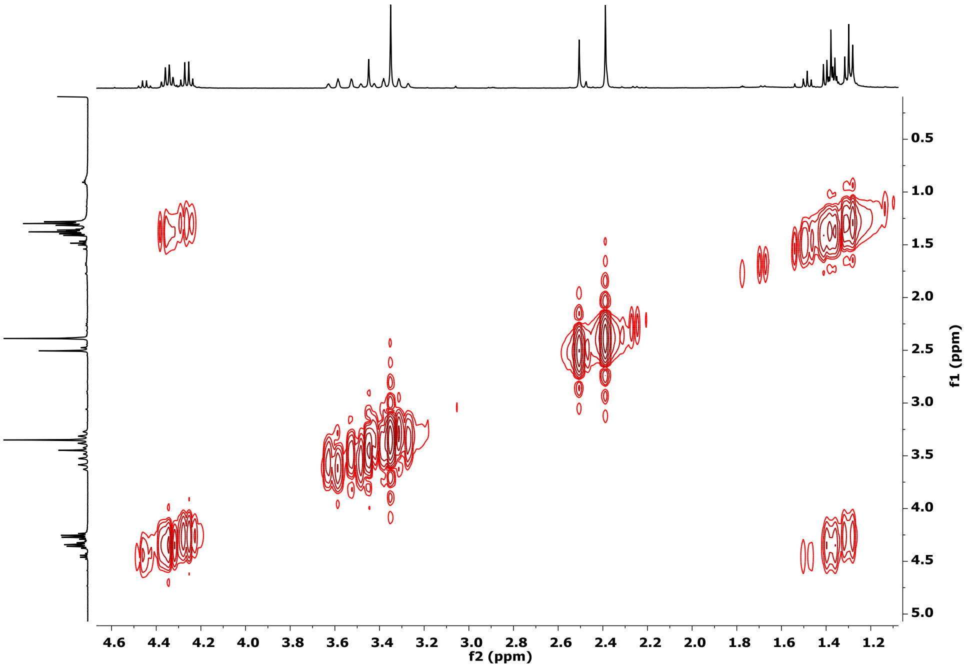

Figure S11. MALDI-TOF HRMS spectrum of **2b**.

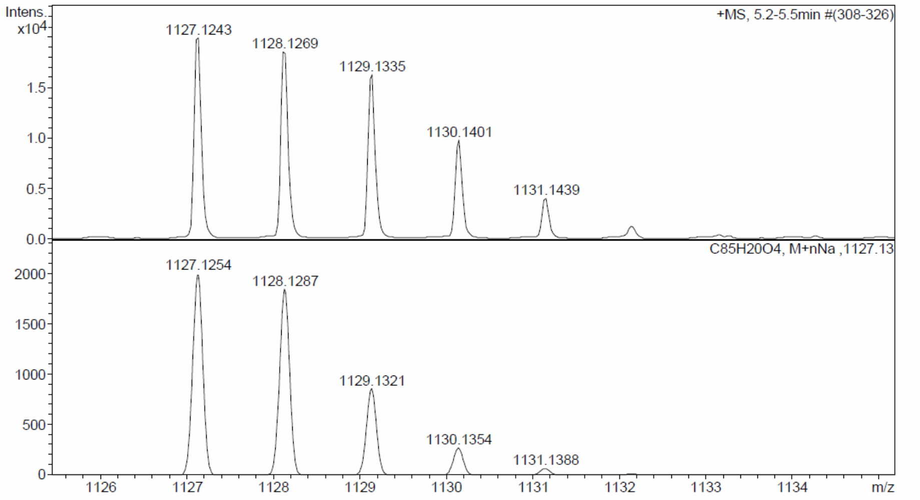

Figure S12. UV–vis spectrum ($CHCl_3$) of compound **2b** (inset: HPLC trace of **2b**).

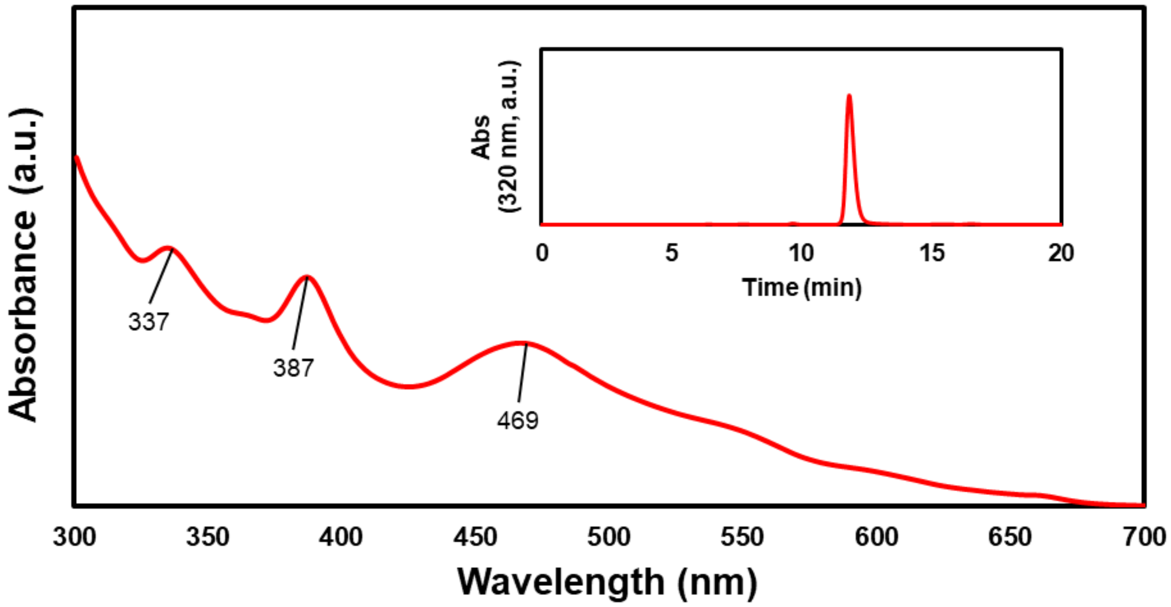

Scheme S3. Oxidative cleavage of **2a**

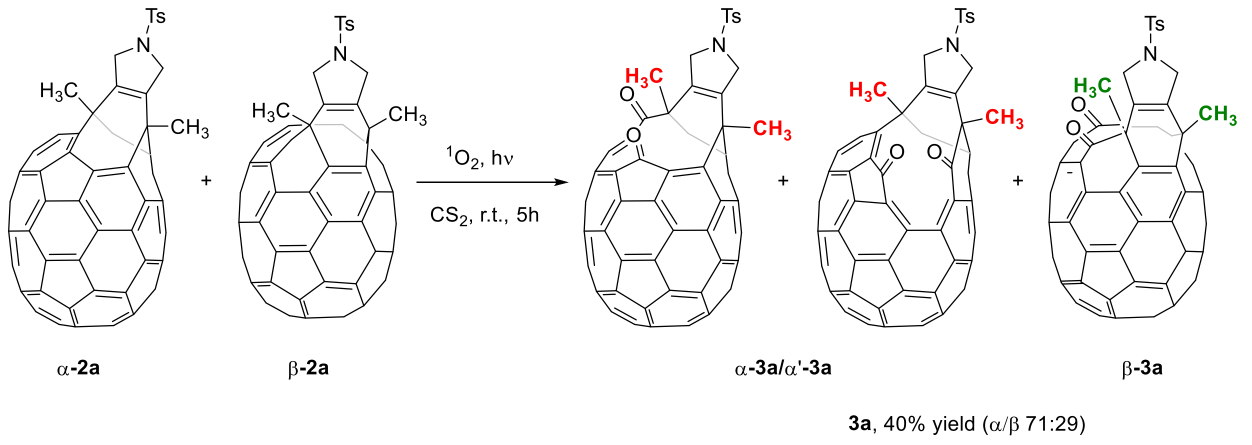

In a 250 mL round-bottomed flask compound **2a** (200 mg, 0.18 mmol) was dissolved in CS_2 (100 mL) and irradiated with a conventional lamp for 5 h (TLC monitoring). The solvent was removed under reduced pressure and the crude product was subjected to column chromatography (SiO_2, 40–63 μm, CS_2/toluene 1:1 → toluene) to provide compound **3a** (83 mg, 40%) as a 56:29:15 mixture of three different isomers as estimated by ^1H NMR integration.

MW ($C_{85}H_{17}NO_4S$): 1148.13 g/mol; **R*f***: 0.13 (toluene); **UV-vis (toluene) λ_{max} (nm)**: 371, 451, 677; **ESI-HRMS (*m/z*)** calcd for $[M+Na]^+$ = 1170.0770; found: 1170.0756.

Figure S13. ^{1}H NMR spectrum (400 MHz, $CDCl_3/CS_2$) of compound **3a**.

a)

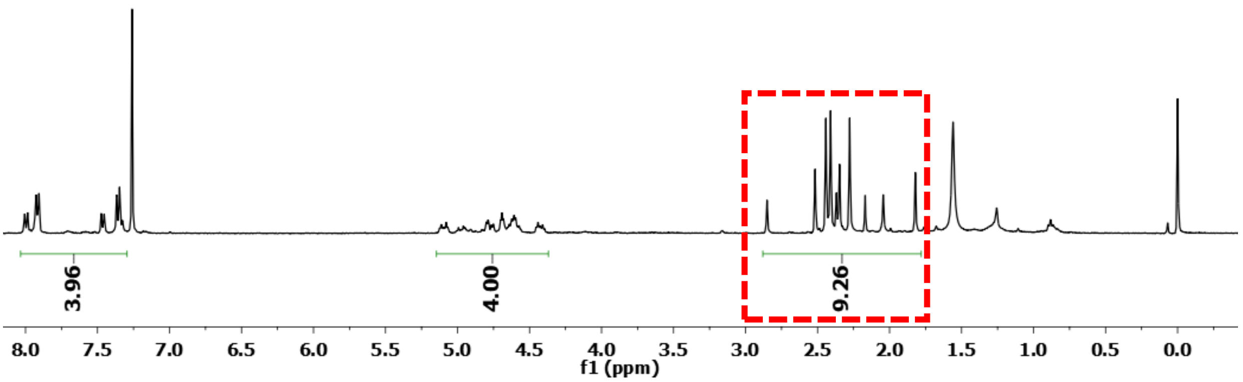

b)

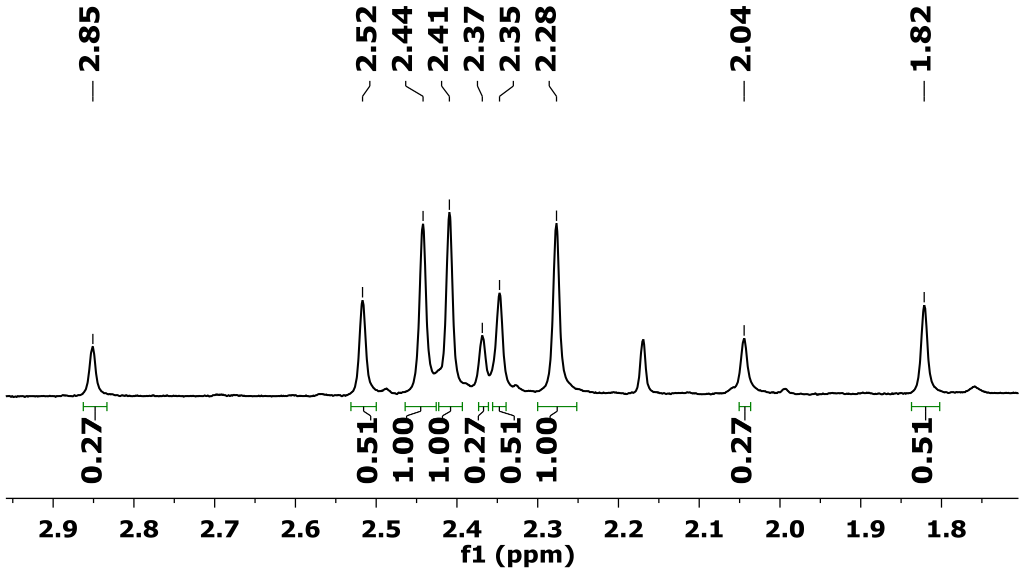

Figure S14. ^{13}C NMR spectrum (101 MHz, $CDCl_3/CS_2$) of compound **3a**.

a)

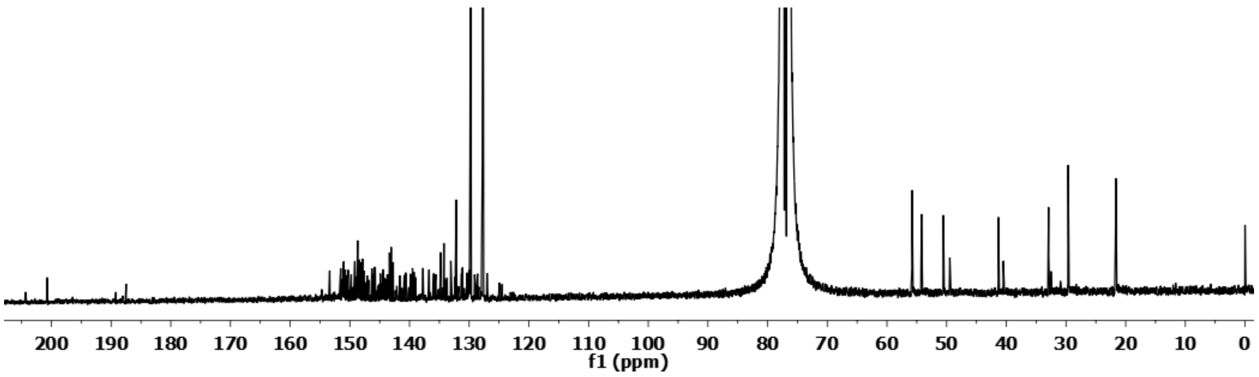

b)

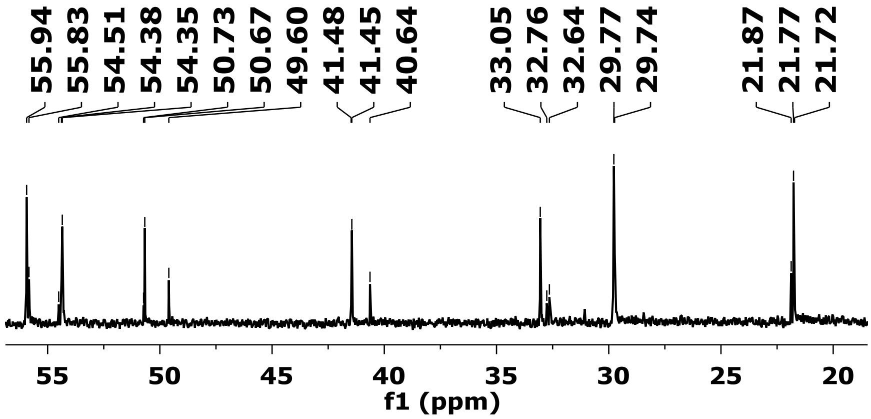

c)

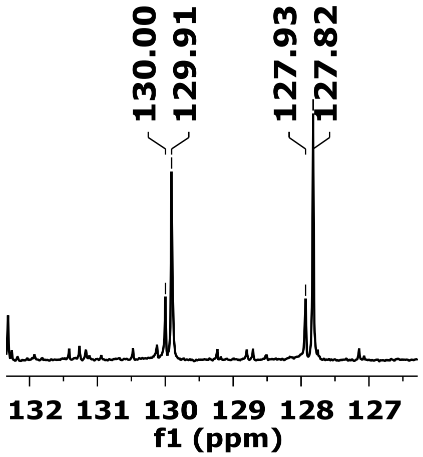

d)

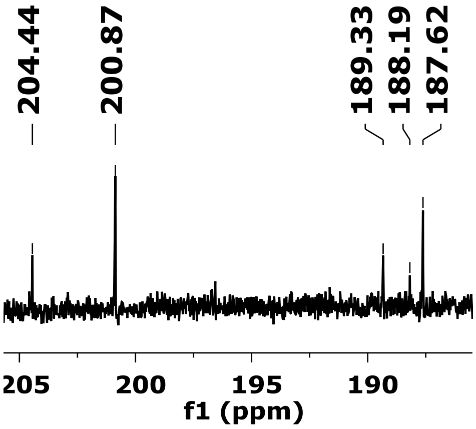

Figure S15. 2D-HSQC NMR spectrum of compound **3a**.

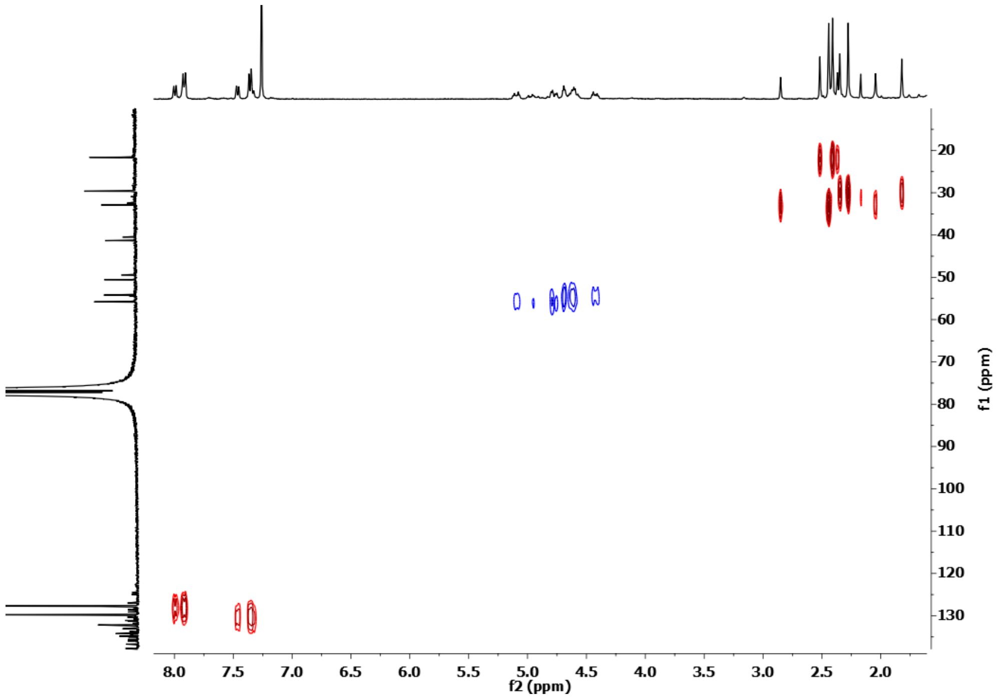

Figure S16. 2D-HMBC NMR spectrum of compound **3a**.

a)

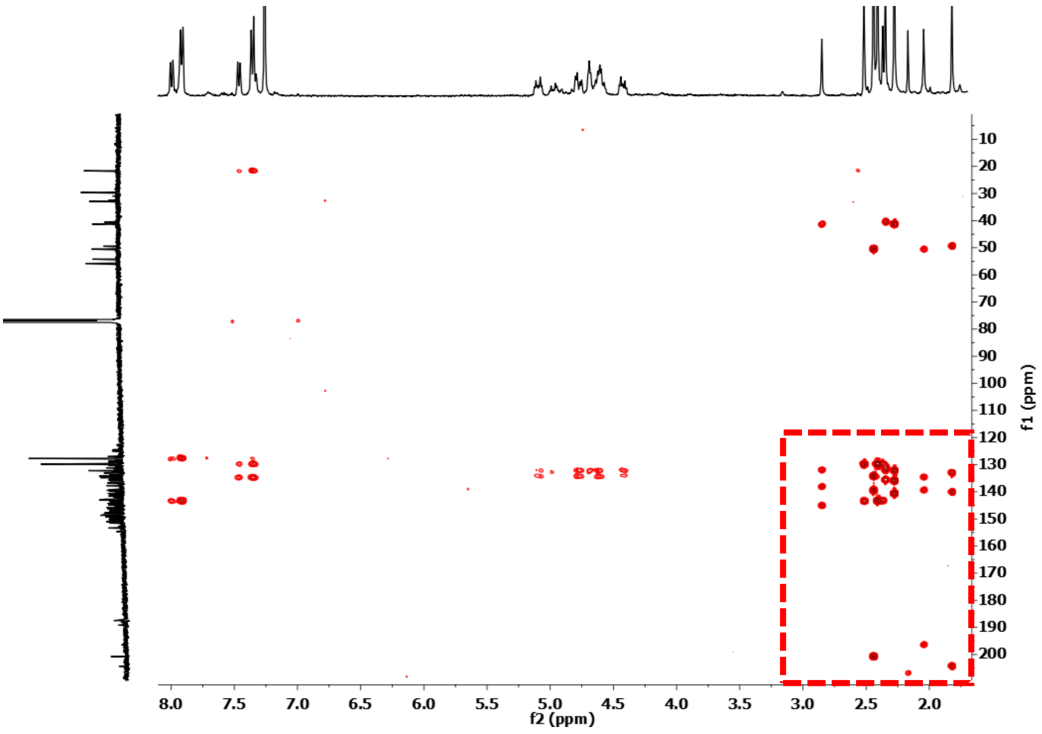

b)

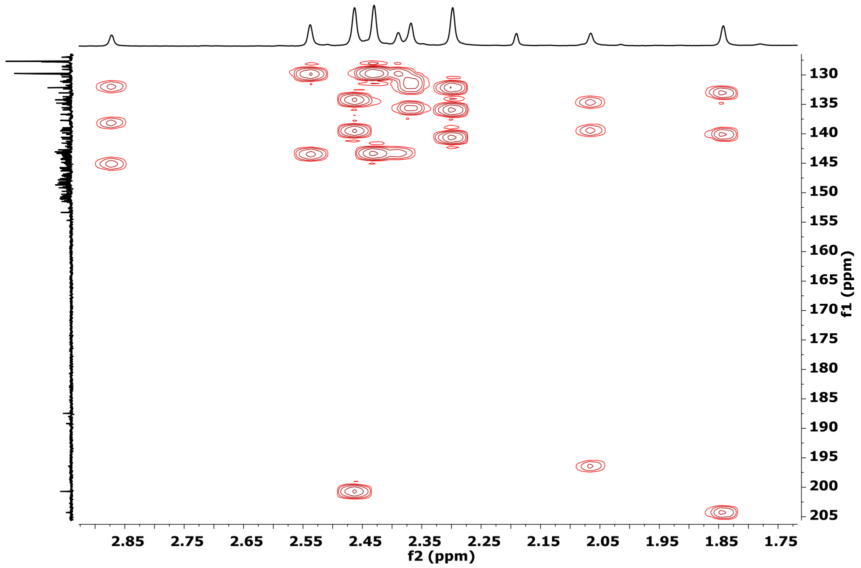

Figure S17. MALDI-TOF HRMS spectrum of compound **3a** (*m/z*).

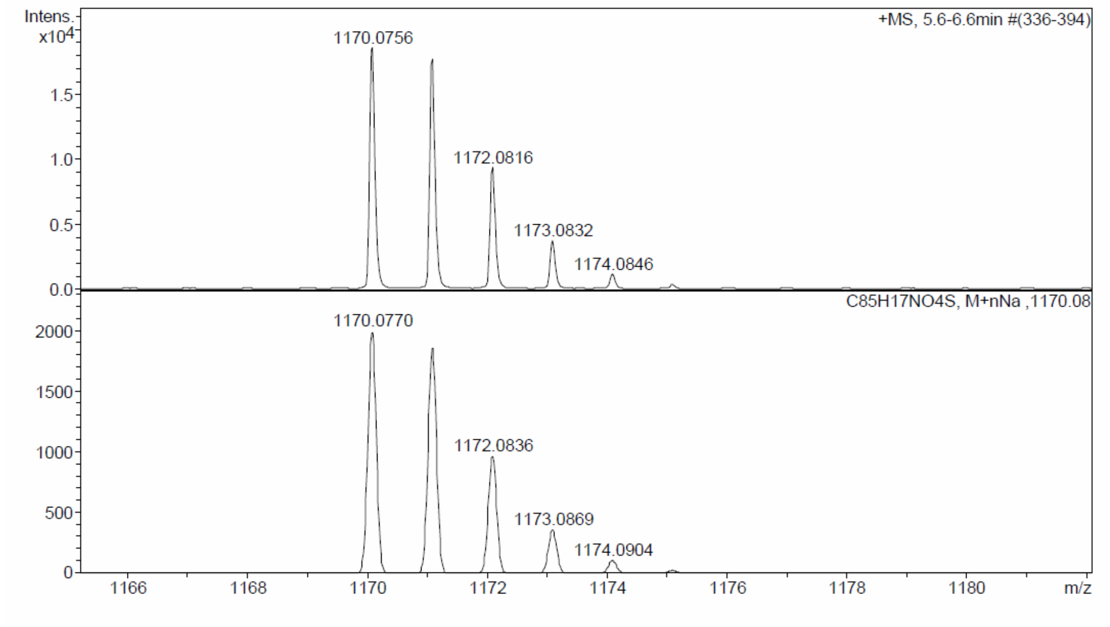

Figure S18. UV–vis spectrum ($CHCl_3$) of compound **3a**.

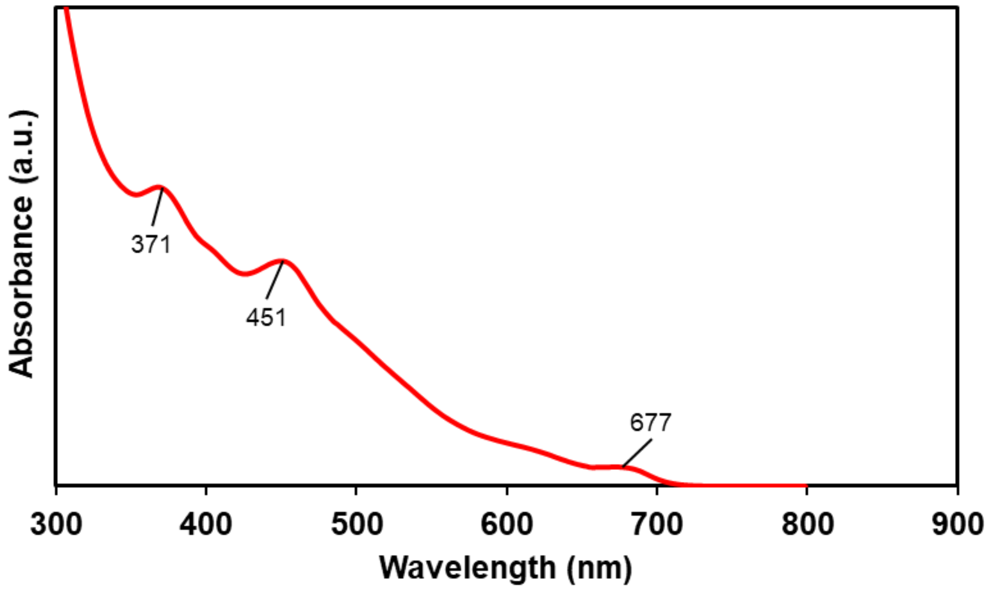

Scheme S4. Preparation and characterization of **3b**.

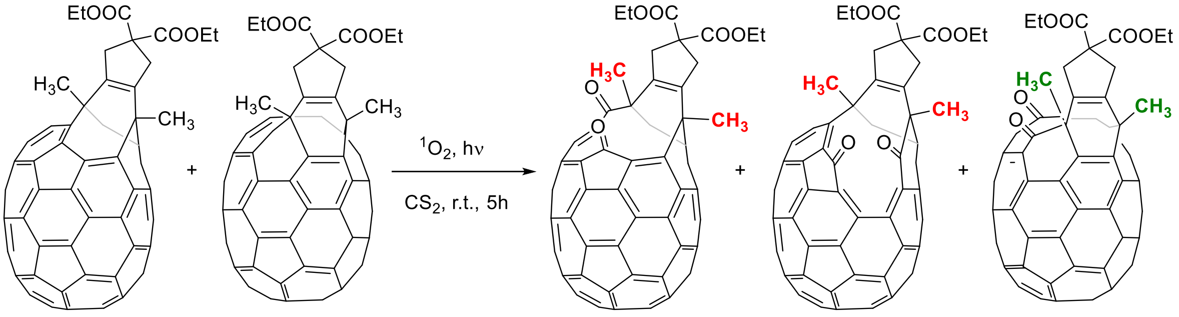

α-**2b** β-**2b** α-**3b**/α'-**3b** β-**3b**

3b, 53% yield (α/β 90:10)

In a 250 mL round-bottomed flask compound **2b** (95 mg, 0.084 mmol) was dissolved in CS_2 (100 mL) and irradiated with a conventional lamp for 5 h (TLC monitoring). The solvent was removed under reduced pressure and the crude product was subjected to column chromatography (SiO_2, 40–63 μm, CS_2/toluene 1:1 → toluene) to provide compound **3a** (52 mg, 53%) as a 52:38:10 mixture of three different isomers as estimated by ^{1}H NMR integration.

MW ($C_{85}H_{20}O_6$): 1136.13 g/mol; **R*f***: 0.32 (toluene); **UV-vis (toluene)** λ_{max} **(nm)**: 369, 451, 677; **ESI-HRMS (*m/z*)** calcd for $[M+Na]^+$ = 1159.1152; found: 1159.1147.

Figure S19. ^{1}H NMR spectrum (400 MHz, $CDCl_3$) of **3b**.

a)

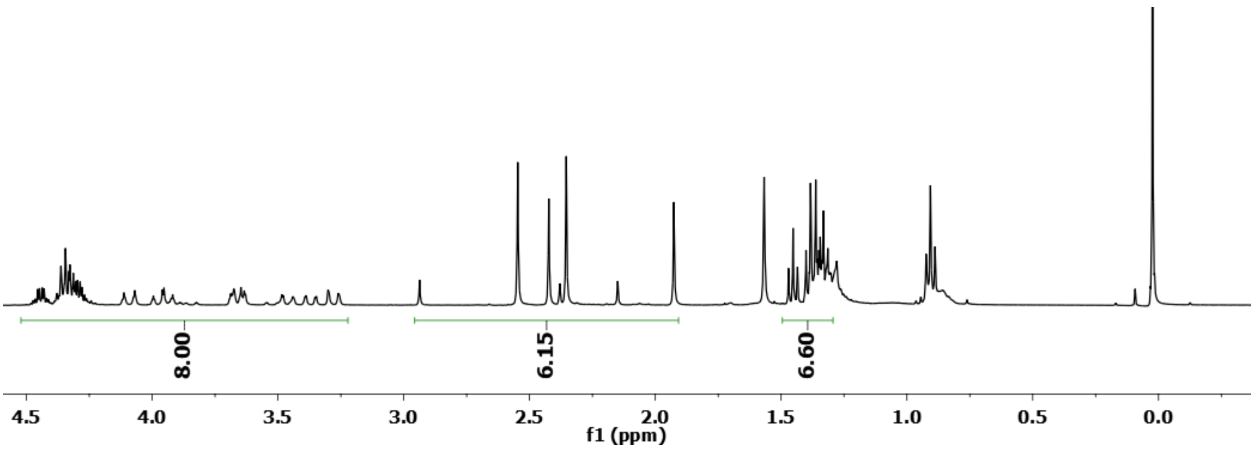

b)

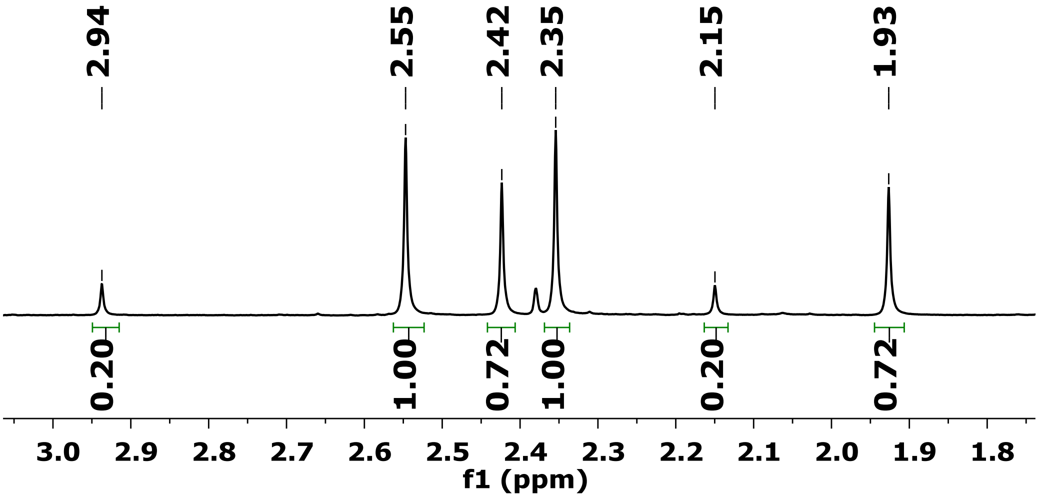

Figure S20. ^{13}C NMR spectrum (101 MHz, $CDCl_3$) of compound **3b**.

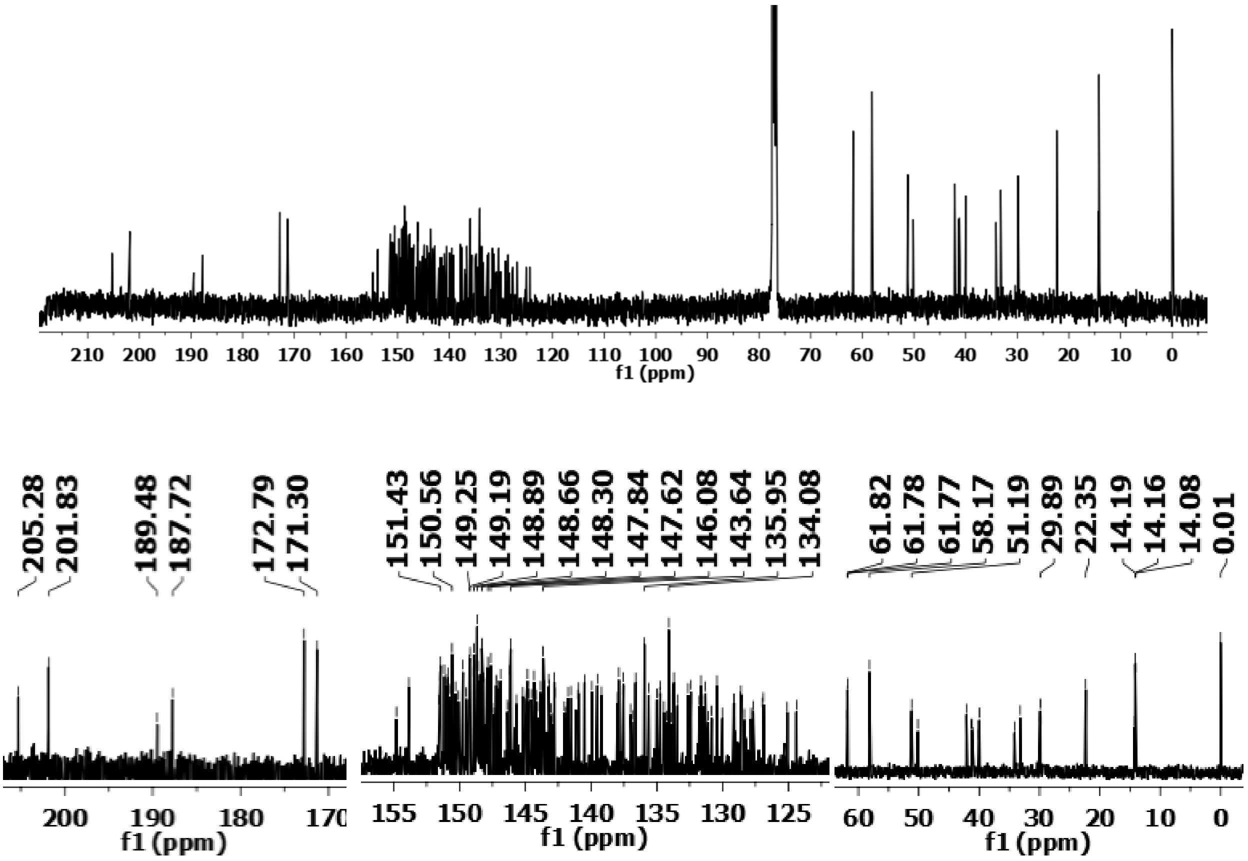

Figure S21. 2D-HSQC NMR spectrum of compound **3b**.

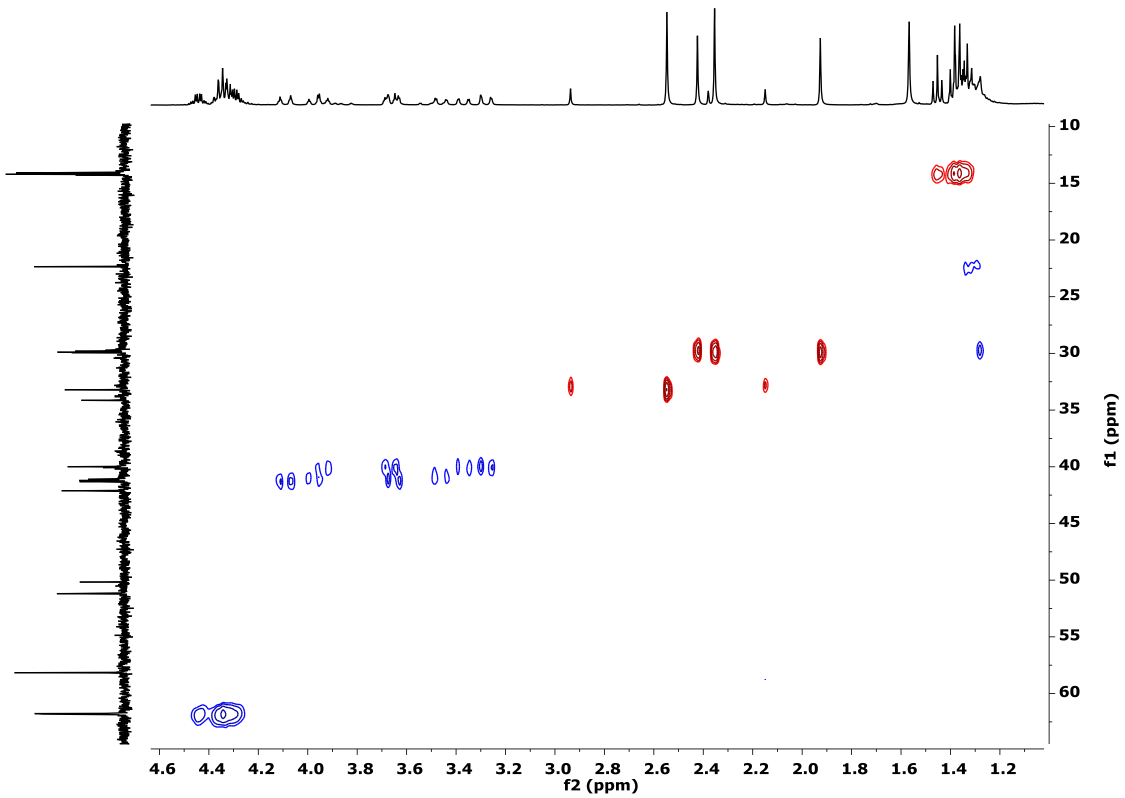

Figure S22. 2D-HMBC NMR spectrum of compound **3b**.

a)

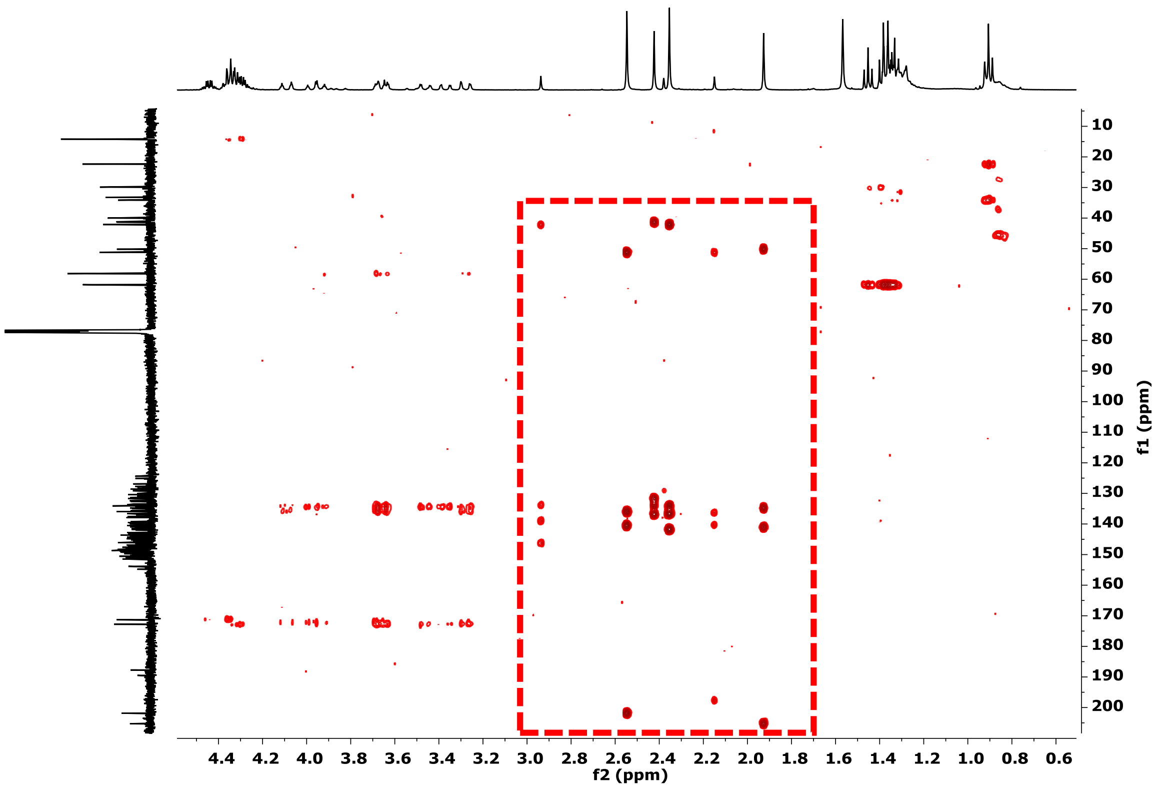

b)

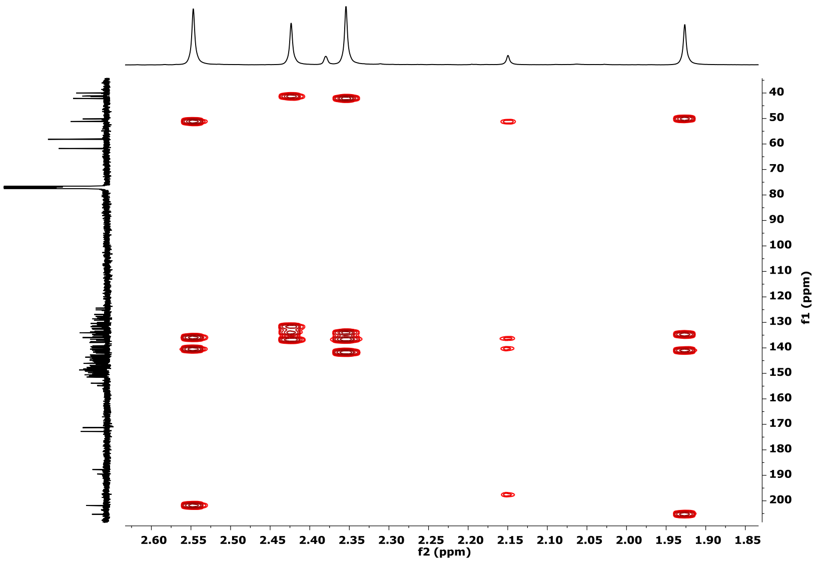

Figure S23. MALDI-TOF HRMS spectrum of compound **3b**.

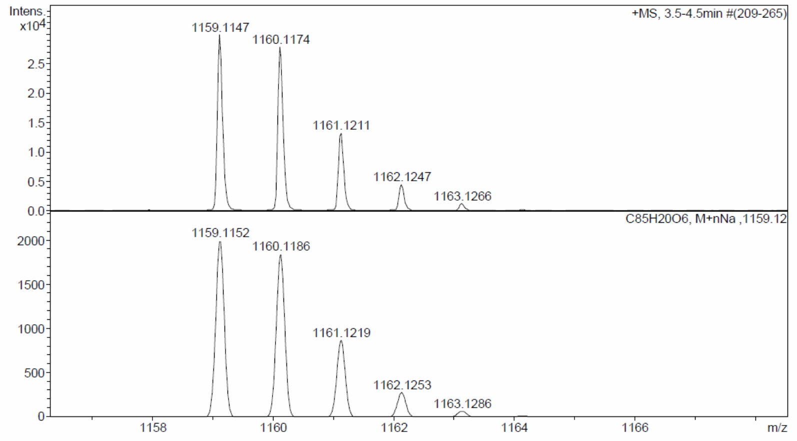

Figure S24. UV–vis spectrum ($CHCl_3$) of compound **3b**.

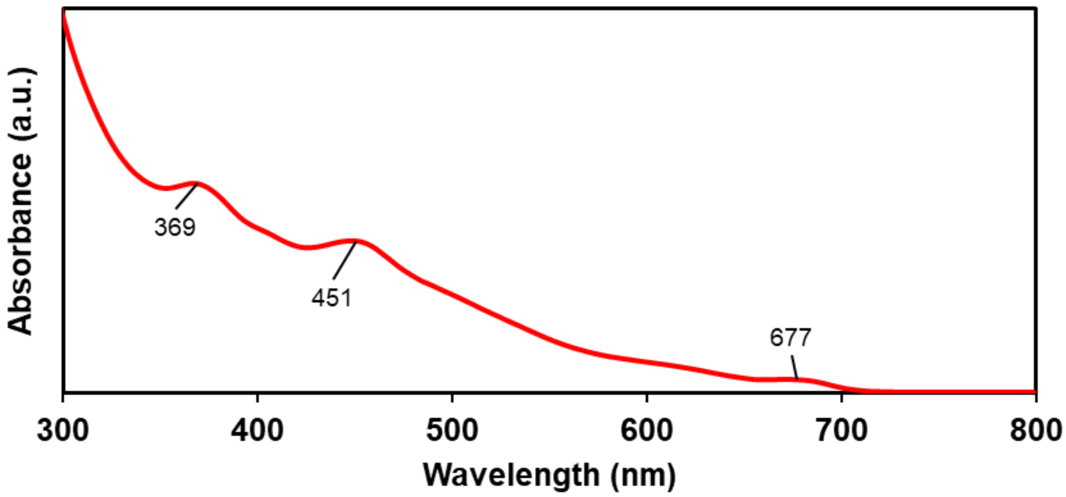

Computational details

Geometries of all stationary points were optimized without symmetry constraints with the Gaussian 09 program [3] using the DFT B3LYP hybrid exchange-correlation functional [4] in conjunction with the all-electron cc-pVDZ basis set [5]. The D3 Grimme energy corrections for dispersion with the original damping function were added. [6] The electronic energy was improved by performing single point energy calculations with the cc-pVTZ basis set [7] and the B3LYP-D3 functional including solvent corrections for *o*-DCB computed with the solvent model based on density (SMD) [8]. Analytical Hessians were computed to determine the nature of stationary points (one and zero imaginary frequencies for TSs and minima, respectively) and to calculate unscaled zero-point energies (ZPEs) as well as thermal corrections and entropy effects using the standard statistical-mechanics relationships for an ideal gas [9]. These two latter terms were computed at 363.15 K and 1 atm to provide the reported relative Gibbs energies. As a summary, the reported Gibbs energies contain electronic energies including solvent effects calculated at the B3LYP-D3/cc-pVTZ//B3LYP-D3/cc-pVDZ level together with gas phase thermal and entropic contributions computed at 363.15 K and 1 atm with the B3LYP-D3/cc-pVDZ method. All stationary points were unambiguously confirmed by IRC calculations. In order to reduce the computational cost, the tosyl substituent in **1a** was substituted by a mesyl substituent in the model substrate and BIPHEP was used as a model phosphine ligand instead of Tol-BINAP.

Figure S25. Molecular structures of the two possible regioisomers of β-**2a** and their relative electronic energies computed at the B3LYP-D3/cc-pVDZ//B3LYP/cc-pVTZ level of theory.

a) **b)**

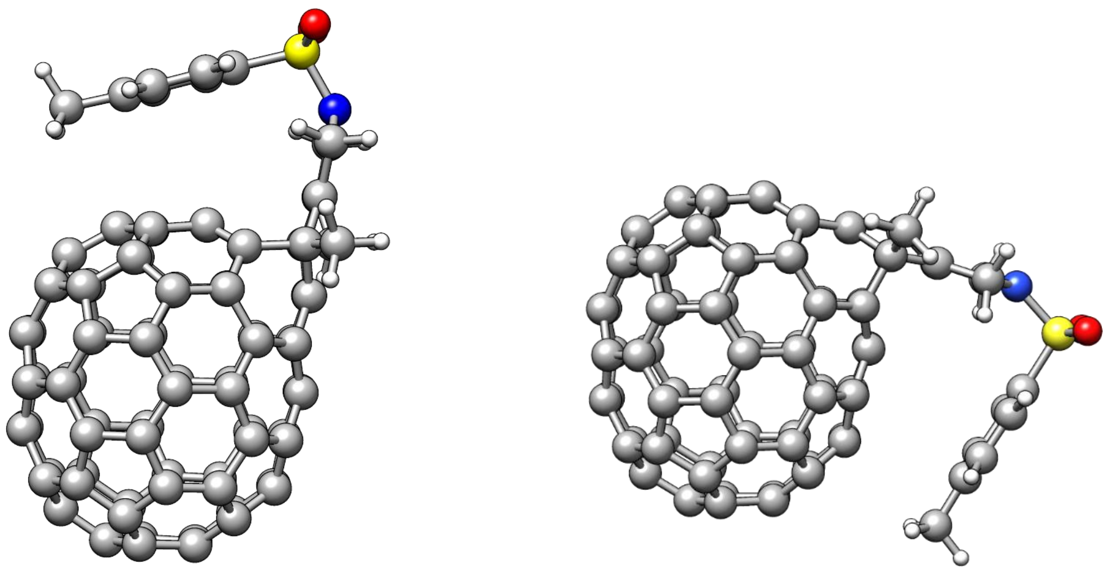

ΔE = 0.0 kcal·mol^{-1} ΔE = 8.8 kcal·mol^{-1}

Figure S26. Molecular structure of **(a)** α**-TS 1 (b)** α**-TS 2 (c)** β**-TS 1 (d)** β**-TS 2**

a)

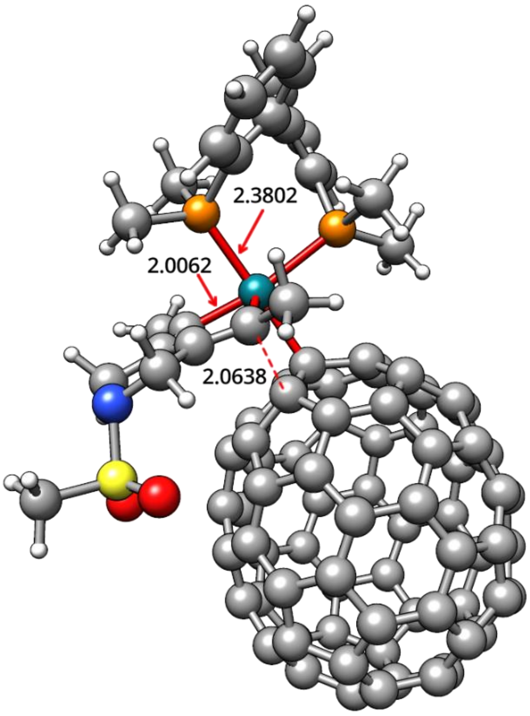

b)

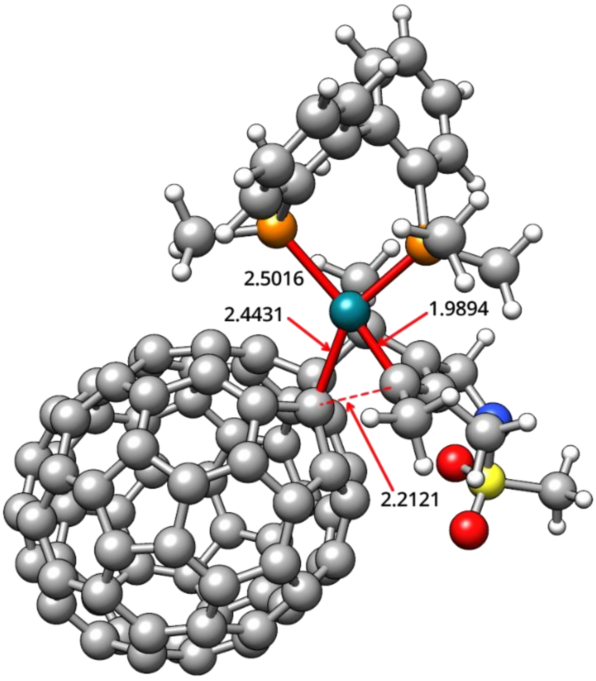

c)

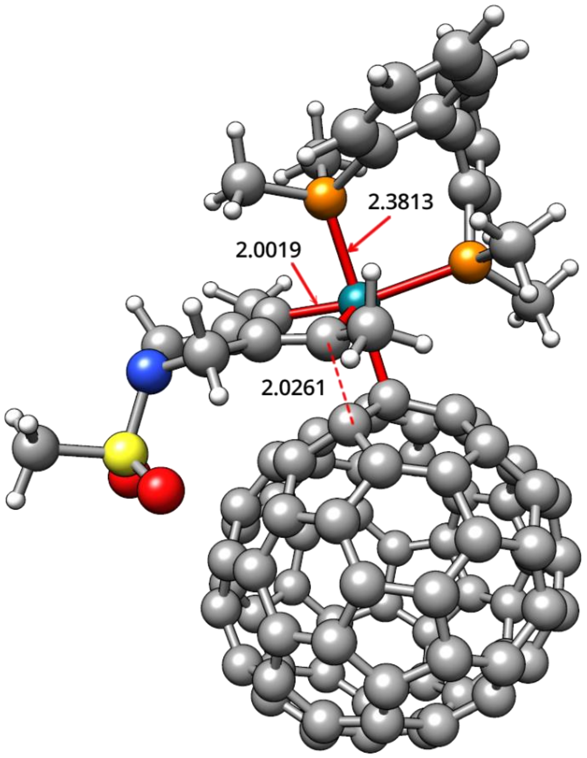

d)

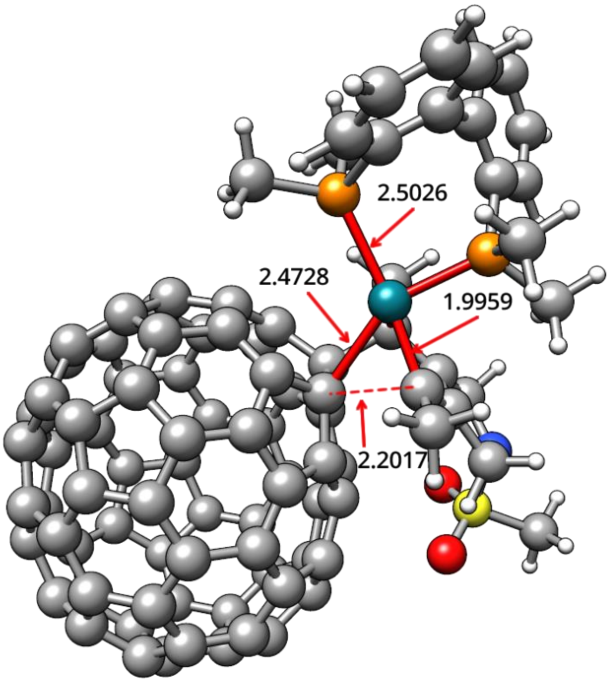

References

[1] Castro, E.; Fernandez-Delgado, O.; Artigas, A.; Zavala, G.; Liu, F.; Moreno-Vicente, A.; Rodríguez-Fortea, A.; Velasquez, J. D.; Poblet, J. M.; Echegoyen, L. *J. Mater. Chem. C* **2020**, *8*, 6813–6819. DOI:10.1039/d0tc01382j.

[2] Artigas, A.; Castanyer, C.; Roig, N.; Lledó, A.; Solà, M.; Pla-Quintana, A.; Roglans, A. *Adv. Synth. Catal.* **2021**, *363*, 3835-3844. DOI: 10.1002/adsc.202100644.

[3] Gaussian 09, Revision E.01, M. J. Frisch, G. W. Trucks, H. B. Schlegel, G. E. Scuseria, M. A. Robb, J. R. Cheeseman, G. Scalmani, V. Barone, B. Mennucci, G. A. Petersson, H. Nakatsuji, M. Caricato, X. Li, H. P. Hratchian, A. F. Izmaylov, J. Bloino, G. Zheng, J. L. Sonnenberg, M. Hada, M. Ehara, K. Toyota, R. Fukuda, J. Hasegawa, M. Ishida, T. Nakajima, Y. Honda, O. Kitao, H. Nakai, T. Vreven, J. A. Montgomery, Jr., J. E. Peralta, F. Ogliaro, M. Bearpark, J. J. Heyd, E. Brothers, K. N. Kudin, V. N. Staroverov, T. Keith, R. Kobayashi, J. Normand, K. Raghavachari, A. Rendell, J. C. Burant, S. S. Iyengar, J. Tomasi, M. Cossi, N. Rega, J. M. Millam, M. Klene, J. E. Knox, J. B. Cross, V. Bakken, C. Adamo, J. Jaramillo, R. Gomperts, R. E. Stratmann, O. Yazyev, A. J. Austin, R. Cammi, C. Pomelli, J. W. Ochterski, R. L. Martin, K. Morokuma, V. G. Zakrzewski, G. A. Voth, P. Salvador, J. J. Dannenberg, S. Dapprich, A. D. Daniels, O. Farkas, J. B. Foresman, J. V. Ortiz, J. Cioslowski, and D. J. Fox, Gaussian, Inc., Wallingford CT, **2013**.

[4] a) Stephens, P. J.; Devlin, F. J.; Chabalowski, C. F.; Frisch, M. J. *J. Phys. Chem.* **1994**, *98*, 11623–11627. doi:10.1021/j100096a001 b) Becke, A. D. *J. Chem. Phys.* **1993**, *98*, 5648–5652. DOI:10.1063/1.464913. c) Lee, C.; Yang, W.; Parr, R. G. *Phys. Rev. B*, **1988**, *37*, 785–789. DOI:10.1103/physrevb.37.785.

[5] Dunning, T. H. *J. Chem. Phys.* **1989**, *90*, 1007–1023. DOI:10.1063/1.456153.

[6] Grimme, S.; Antony, J.; Ehrlich, S.; Krieg, H. *J. Chem. Phys.* **2010**, *132*, 154104. DOI:10.1063/1.3382344.

[7] Kendall, R. A.; Dunning, T. H., Jr.; Harrison, R. J. *J. Chem. Phys.* **1992**, *96*, 6796-6806. DOI:10.1063/1.462569.

[8] Marenich, A. V.; Cramer, C. J.; Truhlar, D. G. *J. Phys. Chem. B* **2009**, *113*, 6378–6396. DOI:10.1021/jp810292n.

[9] P. Atkins, J. De Paula, The Elements of Physical Chemistry, 3rd ed.; Oxford University Press: Oxford, **2006**.
